# Synthesis, resolution and crystal structures of two enanti­omeric rhodamine derivatives

**DOI:** 10.1107/S2056989017001451

**Published:** 2017-02-03

**Authors:** Clifton J. Stephenson, Joel T. Mague, Nathaniel Kamm, Nathalie Aleman, Dayla Rich, Quynh-Nhu Dang, Ha Van Nguyen

**Affiliations:** a Department of Chemistry, Loyola University, New Orleans, LA 70118, USA; b Department of Chemistry, Tulane University, New Orleans, LA 70118, USA

**Keywords:** crystal structure, rhodamine, xanthene, hydrogen bond, chiral sensors

## Abstract

The title mol­ecule, *rac*-6′-bromo-3′-di­ethyl­amino-3*H*-spiro­[2-benzo­furan-1,9′-xanthen]-3-one, C_24_H_20_BrNO_3_, was synthesized and the two enanti­omers which formed were separated. The structures of all three compounds were determined and compared with those of a variety of related derivatives. A notable feature is the fold of the xanthene portion which ranges from 15.15 (13)° in the racemate to 2.42 (2)° in one mol­ecule of the *R* enanti­omer with that for the *S* enanti­omer having an inter­mediate value. The differences are attributed to the number and severity of inter­molecular inter­actions which include C—H⋯O hydrogen bonds, C—H⋯π(ring) and, in the *S* enanti­omer, a π-stacking inter­action between the carbonyl group and an aromatic ring.

## Abstract 2

The synthesis of *rac*-6′-bromo-3′-di­ethyl­amino-3*H*-spiro­[2-benzo­furan-1,9′-xanthen]-3-one and its resolution into separate enanti­omers is described. The structures of the racemate and of the individual enanti­omers were determined and showed differing degrees of folding of the xanthene portion, which were attributed primarily to packing inter­actions. The supra­molecular features of the three structures show significant differences.

## Chemical context   

The compounds synthesized here are part of ongoing work to form chiral sensors based on the supra­molecular inter­actions of chiral rhodamine derivatives with analytes. Enanti­omeric sensing is critical for the efficient and safe formation of chiral pharmaceuticals (LaPlante *et al.*, 2011 ▸) since enanti­omers may have vastly different biological effects including toxicity (Reist *et al.*, 1998 ▸). Most current methods for the detection of enanti­omeric purity involve chromatographic techniques that require costly instrumentation (Wang *et al.*, 2006 ▸). Chiral supra­molecular sensors offer an inexpensive alternative (Chen *et al.*, 2012 ▸; Jo *et al.*, 2014 ▸; Zhang *et al.*, 2014 ▸; Yu & Pu, 2015 ▸). Supra­molecular sensors, such as modified rhodamine derivatives, have garnered recent inter­est as sensors with biological applications (Pak *et al.*, 2015 ▸; You *et al.*, 2015 ▸). Additionally, recent work has shown that rhodamine B can function as a sensor differentiating between diastereomers (Shimizu & Stephenson, 2010 ▸). Herein, we report the synthesis, resolution and structures of two asymmetric rhodamine derivatives **4** and **5** which are being investigated for potential as chiral sensors.
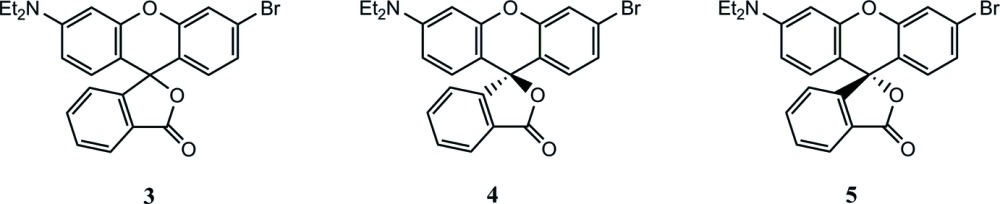
 
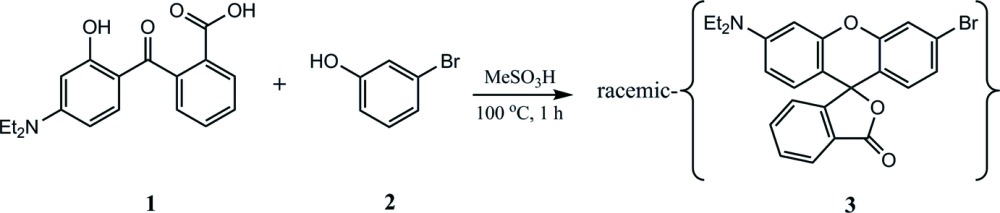


## Structural commentary   

**Figure 1:**
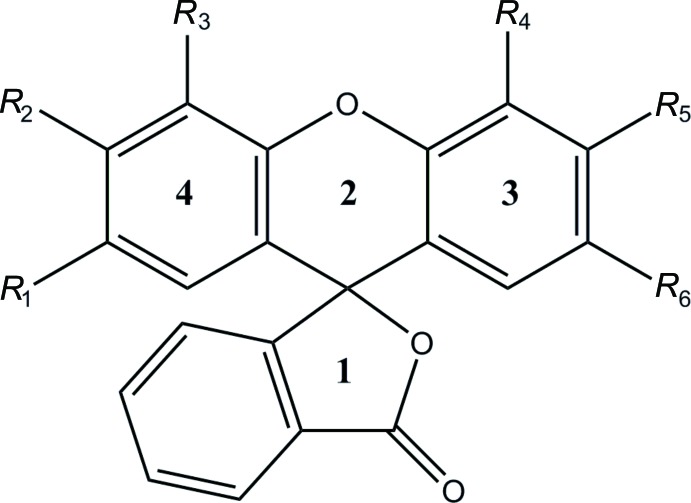
Key for Table 1 ▸.

**Figure 2:**
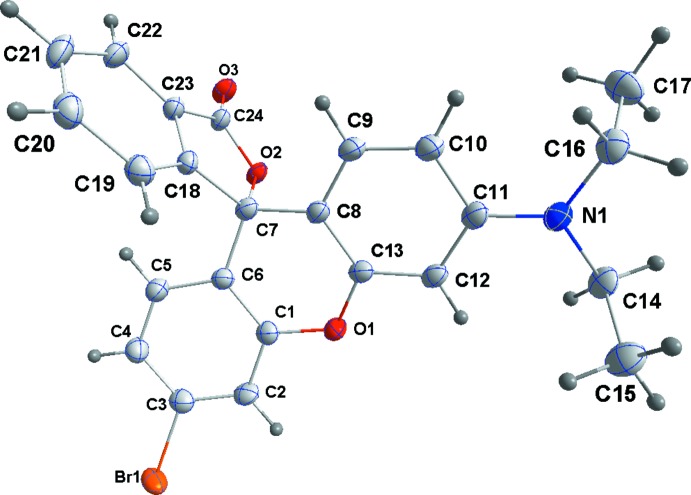
Perspective view of **3**, with the atom-numbering scheme and 50% probability displacement ellipsoids.

**Figure 3:**
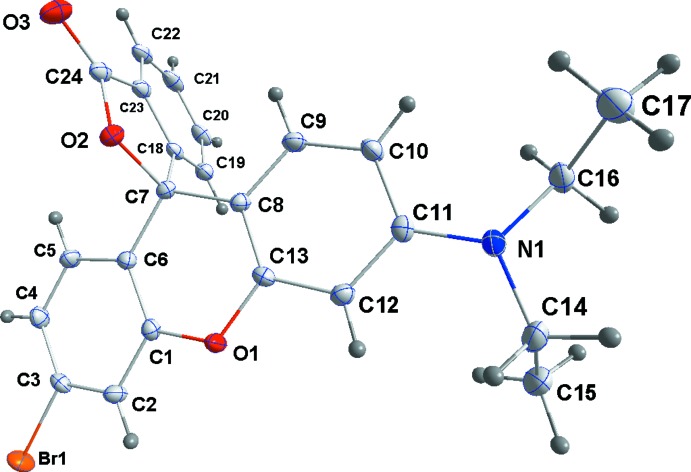
Perspective view of **4**, with the atom-numbering scheme and 50% probability displacement ellipsoids.

In general terms, the structures of **3**–**5** are similar to those of other rhodamine derivatives that have been reported in that the xanthene portion is modestly folded along the O⋯C axis of the central ring and the benzo­furan­one unit is nearly perpendicular to the mean plane of the xanthene unit. Of note in the present work is the variation in the fold of the xanthene portion which is largest in **3**, distinctly smaller in **4** and smallest in **5** but with a significant difference in this angle between the two independent mol­ecules (see the first four entries in Table 1 ▸Fig. 1 ▸). We attribute these differences to the different packing modes for the three structures. In **3** (Fig. 2 ▸), the mol­ecules form zigzag stacks with each pair of adjacent mol­ecules related by centers of symmetry. This leads to pairwise H17*C*⋯C4 separations of 3.04 Å which are only 0.14 Å less than the sum of the van der Waals radii. Were the xanthene portions flatter, these would develop into significant inter­molecular contacts. With **4** and **5** (Figs. 3 ▸ and 4 ▸) in the non-centrosymmetric space group *P*2_1_2_1_2_1_, this stacking is no longer possible and while in **4** there is a van der Waals contact of 2.90 Å between H17*A* and C4^i^ [symmetry code: (i) 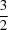 − *x*, 1 − *y*, −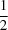 + *z*] which could be lessened by a greater folding, this is opposed by a H2⋯H19^ii^ [symmetry code: (ii) −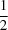 + *x*, 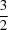 − *y*, 1 − *z*] separation of 2.48 (4) Å which is only 0.08 Å greater than the sum of the van der Waals radii. In the case of **5**, the C8–C13 ring experiences the opposing contacts H40*B*⋯C13 (2.79 Å) and H41*B* ^iii^⋯C11 [2.79 Å; symmetry code: (iii) 1 + *x*, *y*, *z*], both of which are 0.11 Å less than a van der Waals contact and serve to hold this ring in position in the packing. On the other side of this xanthene moiety there is a Br1⋯O6^iv^ [symmetry code: (iv) 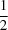 + *x*, 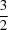 − *y*, 1 − *z*] contact of 3.251 (3) Å which is 0.12 Å less than a van der Waals contact and imparts more of a twist than a simple fold to this portion. This can be seen from the dihedral angle of 5.7 (2)° between the C1–C6 ring and the C1/C6/C7/O1 plane. For the second mol­ecule, there are no short inter­molecular contacts with either side of the xanthene moiety to influence its conformation.

## Supra­molecular features   

**Figure 5:**
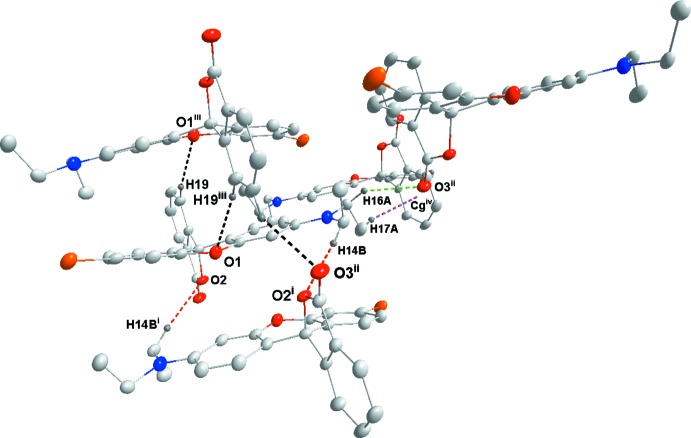
Detail of the inter­molecular inter­actions in **3** with C19—H19⋯O1^iii^, C14—H14*B*⋯O2^i^, and C16—H16*A*⋯O3^ii^ hydrogen bonds shown, respectively, as black, red and green dotted lines, while the C17—H17*A*⋯*Cg* ^iv^ inter­action is given by a purple dotted line. [Symmetry codes: (i) *x*, *y*, −1 + *z*; (ii) −*x*, 1 − *y*, 1 − *z*; (iii) 1 − *x*, 1 − *y*, 1 − *z*; (iv) 1 − *x*, 2 − *y*, 1 − *z*; *Cg* is the centroid of the indicated ring.]

**Figure 6:**
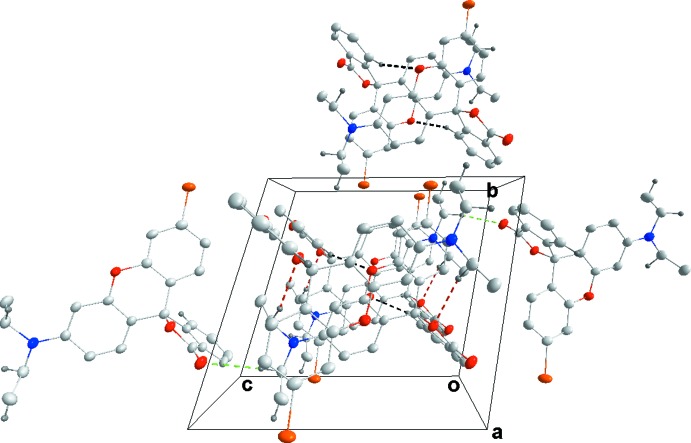
Packing of **3**, viewed along the *a*-axis direction, with the color code for C—H⋯O inter­actions as in Fig. 5 ▸.

**Figure 7:**
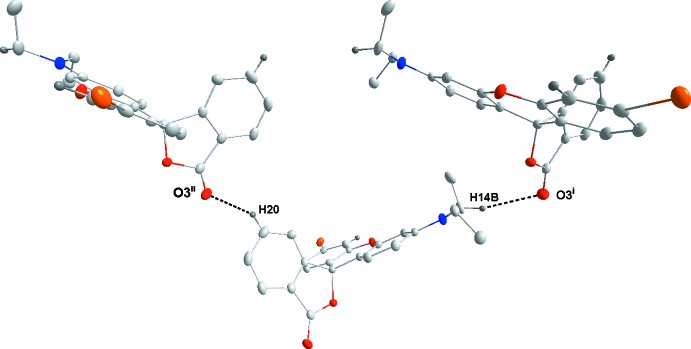
Detail of the inter­molecular inter­actions in **4**. [Symmetry codes: (v) 2 − *x*, 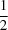 + *y*, 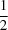 − *z*; (vi) 2 − *x*, 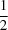 + *y*, 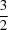 − *z*.]

**Figure 8:**
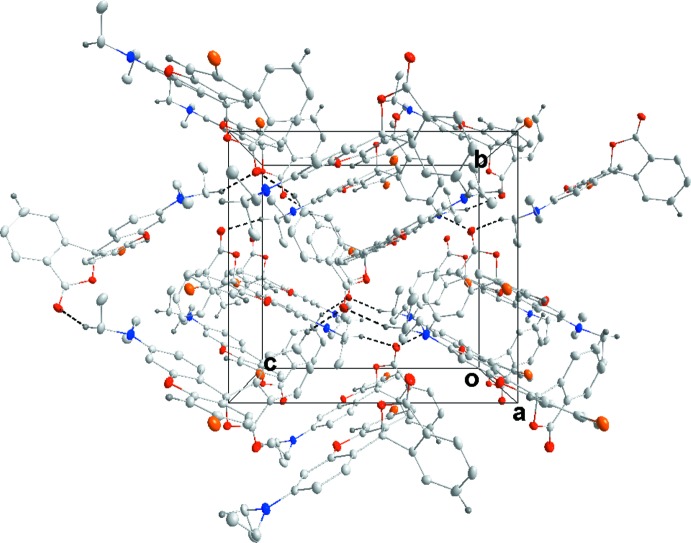
Packing of **4**, viewed along the *a*-axis direction, with C—H⋯O hydrogen bonds shown as dotted lines.

**Figure 9:**
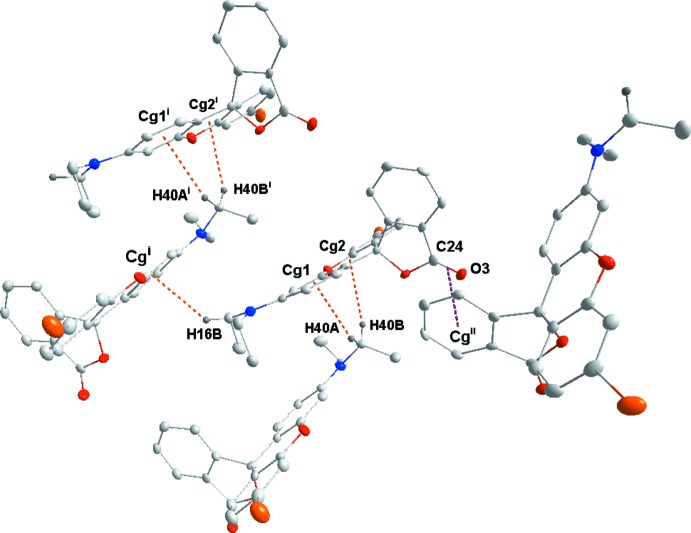
Detail of the inter­molecular inter­actions in **5**. [Symmetry codes: (vii) 1 + *x*, *y*, *z*; (viii) −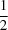 + *x*, 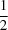 − *y*, 1 − *z*.]

**Figure 10:**
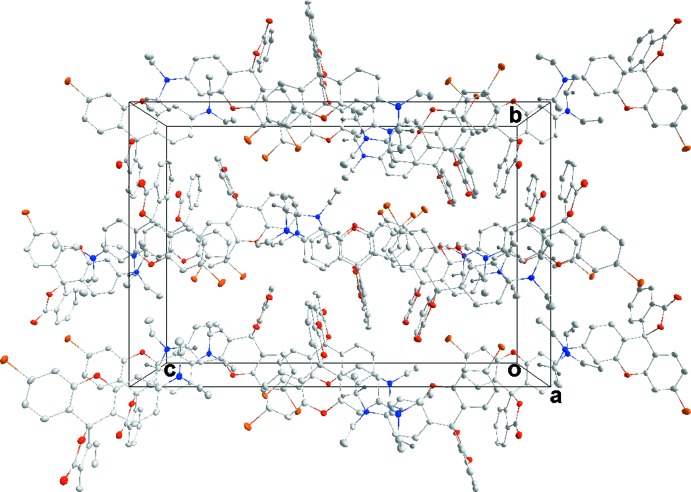
Packing of **5**, viewed along the *a*-axis direction.

Fig. 5 ▸ illustrates the inter­molecular inter­actions in the crystal of **3** with numerical details given in Table 2 ▸. These include two sets of pairwise C—H⋯O hydrogen bonds, two additional sets of C—H⋯O hydrogen bonds and a set of C—H⋯π(ring) inter­actions. The C14⋯H14*B*⋯O2^i^ and C19—H19⋯O1^iii^ inter­actions bind the mol­ecules into stacks along the *a*-axis direction while the C16—H16*A*⋯O3^i^ and C17—H17*A*⋯π(ring)^iv^ inter­actions tie the stacks together (Fig. 6 ▸). Inter­molecular inter­actions are much fewer in the crystal of **4** with C14—H14*B*⋯O3^v^ and C20—H20⋯O3^vi^ hydrogen bonds (Table 3 ▸) forming zigzag chains (Fig. 7 ▸) running approximately along the *c*-axis direction and arranged to form rectangular channels along the *a*-axis direction (Fig. 8 ▸). In the crystal of **5**, the two independent mol­ecules are associated through C40—H40*A*⋯*Cg*1 and C40—H40*B*⋯*Cg*2 inter­actions with these units tied together on one side by C16—H16*B*⋯*Cg* ^i^ inter­actions (Table 4 ▸) and on the other by a π–π inter­action between the C24=O3 bond and the (C18–C23)^ii^ ring [Fig. 9 ▸, centroid–centroid distance = 3.349 (3) Å, angle of C=O vector to centroid = 99.5 (3)°]. The result is a more open 3D structure for this enanti­omer (Fig. 10 ▸).

## Database survey   

There are 71 structures of rhodamine derivatives in the literature, although many are considerably more substituted than **4** and **5** and include a variety of fused-ring systems. Table 1 ▸ lists, in addition to those reported here, 20 other structures which are most nearly comparable to those of this work. In all of these, the lactone ring (ring 1, Fig. 1 ▸) is nearly perpendicular to the mean plane of the central pyran ring (ring 2, Fig. 1 ▸) with dihedral angles ranging from 87.08 (13) to 90.0 (2)° and with three structures having the lactone ring on a crystallographic mirror (Table 1 ▸). In all cases, the xanthene moiety is folded across the O⋯C axis, with the majority having a dihedral angle between rings 3 and 4 (Fig. 1 ▸) in the range 2.42 (3)–7.36 (5)°, but there are six having angles up to 17.5 (5)° (Table 1 ▸). In this latter group, those with the largest angles involve a twist of the xanthene moiety as well as a fold, and this is seen in both symmetrically and unsymmetrically substituted examples. Inspection of inter­molecular contact calculations indicates that the largest dihedral angles correlate with inter­molecular contacts at or somewhat less than the sums of the relevant van der Waals radii.

## Synthesis and crystallization   

As outlined in the scheme[Chem scheme1], compound **1** (2.00 g, 6.73 mmol) was mixed with compound **2** (1.10g, 6.35 mmol) in 16 mL of methyl­sulfonic acid. The mixture was stirred and heated for 1 h at 373 K. The cooled solution was poured over ice and then extracted with dicholoro­methane. A crude yield of the racemate **3** was obtained. A portion of the crude product (1.343 g) was purified on a flash column with 15% ethyl acetate in hexa­nes followed by 100% ethyl acetate. The fractions containing the product were combined and left in a beaker covered with a tissue and the solvent was allowed to evaporate slowly. After about two weeks, the purified racemate yielded a mixture of long needle-shaped as well as plate-shaped crystals (0.293 g, 0.651 mmol, 21.8% yield). Thin layer chromatography demonstrated that both crystal shapes were the desired product (racemate **3**), but only the needles provided a well-refined structure. The melting point range was found to be 420.6–428.9 K for the needles and 415.9–429.8 K for the plates. An NMR spectrum of compound **3** was also obtained (Figs. S1 and S2).

To separate the enanti­omers a mobile phase of 70% hexa­nes, 29.97% ethanol and 0.03% di­ethyl­amine was used. A 4 mg mL^−1^ solution of the racemic bromo-rhodamine deriv­ative, **3** was dissolved in the mobile phase. A two-pump system, both Shimadzu LC-20AD pumps, was utilized for moving the mobile phase through the column. Pump A pumped hexa­nes and Pump B pumped the mixture of 95% ethanol and 0.5% di­ethyl­amine at a flow rate of 3.0 mL min ^−1^ for a total of 16 minutes. The sample was placed in a Shimadzu SIL-20AC autosampler, which injected 400 µL of the sample into the mobile phase. A Shimadzu CTO-20A oven, set at 298 k, held the ChiralPak AD-H column whose stationary phase is amylose tris (3,5-di­methyl­phenyl­carbamate) coated on 5 µm silica-gel. The compounds were eluted and then detected with a Shimadzu SPD-20A UV photodiode array detector with a deuterium lamp set at 230 nm. Each enanti­omer was collected with a Shimadzu FRC-10A fraction collector. One enanti­omer (**4**) elutes from 11.6-12.8 minutes, and the other (**5**) elutes from 13.4–14.8 minutes using the method described above. Slow evaporation of the solutions of the pure enanti­omers at room temperature afforded X-ray quality crystals over 1-5 days.

## Refinement   

Crystal data, data collection and structure refinement details are summarized in Table 5 ▸. In **3** and **5**, H atoms attached to carbon were placed in calculated positions (C—H = 0.95–0.99 Å) and included as riding contributions with isotropic displacement parameters 1.2–1.5 times those of the attached atoms. In **4**, the methyl group H atoms were placed in calculated positions as in **3** and **5** (due to poor geometry resulting from individual refinement) while the remainder were refined.

## Supplementary Material

Crystal structure: contains datablock(s) global, 3, 4, 5. DOI: 10.1107/S2056989017001451/sj5518sup1.cif

Structure factors: contains datablock(s) 3. DOI: 10.1107/S2056989017001451/sj55183sup2.hkl

Structure factors: contains datablock(s) 4. DOI: 10.1107/S2056989017001451/sj55184sup3.hkl

Structure factors: contains datablock(s) 5. DOI: 10.1107/S2056989017001451/sj55185sup4.hkl

Supporting information file. DOI: 10.1107/S2056989017001451/sj55183sup5.cml

Supporting information file. DOI: 10.1107/S2056989017001451/sj55184sup6.cml

Supporting information file. DOI: 10.1107/S2056989017001451/sj55185sup7.cml

CCDC references: 1529967, 1529966, 1529965

Additional supporting information:  crystallographic information; 3D view; checkCIF report

## Figures and Tables

**Figure 4:**
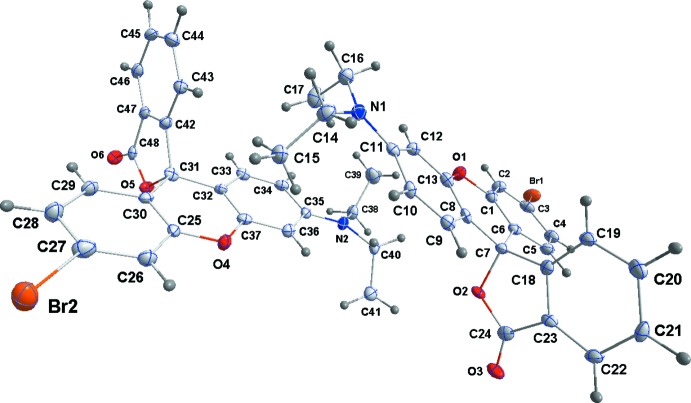
The asymmetric unit of **5**, with the atom-numbering scheme and 50% probability displacement ellipsoids.

**Table 1:** Dihedral angles (°) in selected rhodamine derivatives. *R*_1_–*R*_6_ positions are defined in Fig. 1 ▸.

*R*_1_	*R*_2_	*R*_3_	*R*_4_	*R*_5_	*R*_6_	ring 1–ring 2	ring 3–ring 4	Ref.
H	Et_2_N	H	H	Br	H	88.05 (14)	15.15 (13)	*a*
H	Et_2_N	H	H	Br	H	88.11 (11)	9.74 (11)	*b*
H	Et_2_N	H	H	Br	H	84.2 (2)	6.45 (19)	*c*
H	Et_2_N	H	H	Br	H	89.6 (2)	2.4 (2)	*c*
H	Et_2_N	H	H	Et_2_N	H	89.2 (2)	4.2 (2)	*d*
H	OH	mbz	H	OH	H	88.17 (19)	2.82 (2)	*e*
Cl	OH	CH_2_tm	CH_2_tm	OH	Cl	90^1^	15.0 (3)	*f*
Cl	OH	CH_2_mo	CH_2_mo	OH	Cl	90^1^	7.5 (17)	*f*
H	Et_2_N	H	H	Et_2_N	H	89.59 (5)	7.36 (5)	*g*
H	Et_2_n	H	H	Et_2_N	H	89.58 (5)	4.59 (5)	*g*
H	Et_2_N	H	H	Me	NH(x­yl)	88.8 (14)	3.74 (17)	*h*
H	Et_2_N	H	H	H	NO_2_	89.4 (2)	6.1 (2)	*i*
H	MeO	H	H	OH	H	88.7 (3)	6.3 (3)	*j*
H	Ethm	H	H	Ethm	H	88.64 (17)	14.62 (13)	*k*
NO_2_	Ethm	Br	Br	Ethm	NO_2_	89.7 (4)	17.5 (5)	*k*
H	OH	H	H	OH	H	89.67 (12)	8.19 (11)	*l*
H	OH	H	H	OH	H	90^1^	4.24 (11)	*l*
H	OH	H	H	OH	H	87.30 (7)	6.25 (7)	*l*
H	OH	H	H	OH	H	90.0 (2)	2.4 (2)	*l*
H	OH	CHO	H	OH	H	89.7 (3)	2.5 (3)	*m*
H	OH	CHO	CHO	OH	H	88.47 (13)	4.68 (12)	*m*
H	Bu_2_N	H	H	Me	NHPh	87.08 (13)	13.76 (12)	*n*
H	EtC(O)O	H	H	EtC(O)O	H	89.29 (14)	15.16 (11)	*o*
MeNH_2_	H	H	H	Et_2_N	H	89.1 (3)	6.9 (3)	*p*

^1^Ring 1 lies on a crystallographic mirror. Notes: (*a*) This work (compound **3**); (*b*) this work (compound **4**); (*c*) this work (compound **5**); (*d*) Zhang *et al.* (2015 ▸); (*e*) Hou *et al.* (2012 ▸) (mbz = PhC(O)NHN=CH); (*f*) Swamy *et al.* (2009 ▸) (tm = thio­morpholino; mo = morpholino); (*g*) Kvick *et al.* (2000 ▸) (first line = mol­ecule 1, second line = mol­ecule 2); (*h*) Li *et al.* (2006 ▸) (xyl = 2,4-Me_2_C_6_H_3_); (*i*) Liu *et al.* (1995 ▸); (*j*) Mchedlov-Petrossyan *et al.* (2015 ▸); (*k*) Berscheid *et al.* (1992 ▸) (Ethm = OCH_2_C≡CH); (*l*) Bučar *et al.* (2013 ▸); (*m*) Wang *et al.* (2005 ▸); (*n*) Okada (1996 ▸); (*o*) Wang *et al.* (1990 ▸); (*p*) Miao *et al.* (1996 ▸).

**Table 2:** Hydrogen-bond geometry (Å, °) for **3** [Chem scheme1] *Cg* is the centroid of the C8–C13 ring.

*D*—H⋯*A*	*D*—H	H⋯*A*	*D*⋯*A*	*D*—H⋯*A*
C14—H14*B*⋯O2^i^	0.99	2.68	3.649 (4)	165
C16—H16*A*⋯O3^ii^	0.99	2.64	3.522 (3)	148
C16—H16*B*⋯Br1^iii^	0.99	2.99	3.939 (3)	162
C17—H17*A*⋯*Cg* ^iv^	0.98	2.75	3.666 (4)	156
C19—H19⋯O1^iii^	0.95	2.57	3.485 (4)	161
C20—H20⋯O3^v^	0.95	2.58	3.421 (3)	148

Symmetry codes: (i) 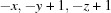; (ii) 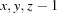; (iii) 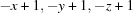; (iv) 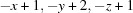; (v) 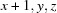.

**Table 3:** Hydrogen-bond geometry (Å, °) for **4** [Chem scheme1]

*D*—H⋯*A*	*D*—H	H⋯*A*	*D*⋯*A*	*D*—H⋯*A*
C14—H14*B*⋯O3^i^	0.99 (2)	2.68 (2)	3.621 (3)	160.7 (19)
C20—H20⋯O3^ii^	0.94 (2)	2.41 (3)	3.163 (3)	138 (3)

Symmetry codes: (i) 
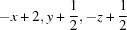
; (ii) 
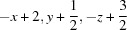
.

**Table 4:** Hydrogen-bond geometry (Å, °) for **5** [Chem scheme1] *Cg*1 and *Cg*2 are the centroids of the C8–C13 and O1,C1,C6,C7,C8,C13 rings, respectively.

*D*—H⋯*A*	*D*—H	H⋯*A*	*D*⋯*A*	*D*—H⋯*A*
C16—H16*B*⋯*Cg* ^i^	0.99	2.81	3.583 (4)	136
C40—H40*A*⋯*Cg*1	0.99	2.79	3.534 (4)	132
C40—H40*B*⋯*Cg*2	0.99	2.83	3.580 (4)	133

Symmetry code: (i) 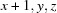.

**Table 5:** Experimental details

	**3**	**4**	**5**
Crystal data			
Chemical formula	C_24_H_20_BrNO_3_	C_24_H_20_BrNO_3_	C_24_H_20_BrNO_3_
*M*_r_	450.32	450.32	450.32
Crystal system, space group	Triclinic, *P* 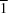	Orthorhombic, *P*2_1_2_1_2_1_	Orthorhombic, *P*2_1_2_1_2_1_
Temperature (K)	150	100	100
*a*, *b*, *c* (Å)	8.3074 (4), 11.1871 (5), 11.7693 (6)	11.0772 (6), 13.0582 (8), 13.8966 (8)	8.1529 (13), 18.185 (3), 26.860 (4)
α, β, γ (°)	102.384 (2), 91.106 (2), 109.581 (2)	90, 90, 90	90, 90, 90
*V* (Å^3^)	1001.60 (8)	2010.1 (2)	3982.3 (11)
*Z*	2	4	8
Radiation type	Cu *K*α	Mo *K*α	Mo *K*α
μ (mm^−1^)	3.01	2.07	2.09
Crystal size (mm)	0.11 × 0.07 × 0.06	0.31 × 0.12 × 0.10	0.26 × 0.06 × 0.04

Data collection			
Diffractometer	Bruker D8 VENTURE PHOTON 100 CMOS	Bruker *SMART* *APEX* CCD	Bruker *SMART* *APEX* CCD
Absorption correction	Multi-scan (*SADABS*; Bruker, 2016 ▸)	Multi-scan (*SADABS*; Bruker, 2016 ▸)	Multi-scan (*SADABS*; Bruker, 2016 ▸)
*T*_min_, *T*_max_	0.59, 0.84	0.69, 0.82	0.70, 0.92
No. of measured, independent and observed [*I* > 2σ(*I*)] reflections	7525, 3725, 3415	39006, 5417, 4926	38240, 10174, 7285
*R*_int_	0.037	0.043	0.075
(sin θ/λ)_max_ (Å^−1^)	0.618	0.687	0.685

Refinement			
*R*[*F* ^2^ > 2σ(*F* ^2^)], *wR*(*F* ^2^), *S*	0.045, 0.124, 1.03	0.026, 0.058, 0.99	0.045, 0.093, 0.97
No. of reflections	3725	5417	10174
No. of parameters	264	320	527
H-atom treatment	H-atom parameters constrained	H atoms treated by a mixture of independent and constrained refinement	H-atom parameters constrained
Δρ_max_, Δρ_min_ (e Å^−3^)	1.01, −0.98	0.62, −0.25	0.92, −0.34
Absolute structure	–	Flack *x* determined using 1981 quotients [(*I* ^+^)−(*I* ^−^)]/[(*I* ^+^)+(*I* ^−^) (Parsons *et al.*, 2013 ▸)	Flack *x* determined using 2575 quotients [(*I* ^+^)−(*I* ^−^)]/[(*I* ^+^)+(*I* ^−^) (Parsons *et al.*, 2013 ▸)
Absolute structure parameter	–	−0.006 (3)	−0.002 (6)

Computer programs: *APEX3* and *SAINT* (Bruker, 2016 ▸), *SHELXT* (Sheldrick, 2015*a* ▸), *SHELXL2014* (Sheldrick, 2015*b* ▸), *DIAMOND* (Brandenburg & Putz, 2012 ▸) and *SHELXTL* (Sheldrick, 2008 ▸).

## supplementary crystallographic information

### (3) rac-6'-Bromo-3'-diethylamino-3H-spiro[2-benzofuran-1,9'-xanthen]-3-one Crystal data

**Table:**

C_24_H_20_BrNO_3_	*Z* = 2
*M**_r_* = 450.32	*F*(000) = 460
Triclinic, *P*1	*D*_x_ = 1.493 Mg m^−^^3^
*a* = 8.3074 (4) Å	Cu *K*α radiation, λ = 1.54178 Å
*b* = 11.1871 (5) Å	Cell parameters from 6586 reflections
*c* = 11.7693 (6) Å	θ = 3.9–72.5°
α = 102.384 (2)°	µ = 3.01 mm^−^^1^
β = 91.106 (2)°	*T* = 150 K
γ = 109.581 (2)°	Column, colourless
*V* = 1001.60 (8) Å^3^	0.11 × 0.07 × 0.06 mm

### (3) rac-6'-Bromo-3'-diethylamino-3H-spiro[2-benzofuran-1,9'-xanthen]-3-one Data collection

**Table:**

Bruker D8 VENTURE PHOTON 100 CMOS diffractometer	3725 independent reflections
Radiation source: INCOATEC IµS micro-focus source	3415 reflections with *I* > 2σ(*I*)
Mirror monochromator	*R*_int_ = 0.037
Detector resolution: 10.4167 pixels mm^-1^	θ_max_ = 72.5°, θ_min_ = 3.9°
ω scans	*h* = −10→9
Absorption correction: multi-scan (*SADABS*; Bruker, 2016)	*k* = −13→13
*T*_min_ = 0.59, *T*_max_ = 0.84	*l* = −12→14
7525 measured reflections	

### (3) rac-6'-Bromo-3'-diethylamino-3H-spiro[2-benzofuran-1,9'-xanthen]-3-one Refinement

**Table:**

Refinement on *F*^2^	Primary atom site location: structure-invariant direct methods
Least-squares matrix: full	Secondary atom site location: difference Fourier map
*R*[*F*^2^ > 2σ(*F*^2^)] = 0.045	Hydrogen site location: inferred from neighbouring sites
*wR*(*F*^2^) = 0.124	H-atom parameters constrained
*S* = 1.03	*w* = 1/[σ^2^(*F*_o_^2^) + (0.0814*P*)^2^ + 0.6226*P*] where *P* = (*F*_o_^2^ + 2*F*_c_^2^)/3
3725 reflections	(Δ/σ)_max_ < 0.001
264 parameters	Δρ_max_ = 1.01 e Å^−^^3^
0 restraints	Δρ_min_ = −0.98 e Å^−^^3^

### (3) rac-6'-Bromo-3'-diethylamino-3H-spiro[2-benzofuran-1,9'-xanthen]-3-one Special details

**Table:**

Geometry. All esds (except the esd in the dihedral angle between two l.s. planes) are estimated using the full covariance matrix. The cell esds are taken into account individually in the estimation of esds in distances, angles and torsion angles; correlations between esds in cell parameters are only used when they are defined by crystal symmetry. An approximate (isotropic) treatment of cell esds is used for estimating esds involving l.s. planes.
Refinement. Refinement of F^2^ against ALL reflections. The weighted R-factor wR and goodness of fit S are based on F^2^, conventional R-factors R are based on F, with F set to zero for negative F^2^. The threshold expression of F^2^ > 2sigma(F^2^) is used only for calculating R-factors(gt) etc. and is not relevant to the choice of reflections for refinement. R-factors based on F^2^ are statistically about twice as large as those based on F, and R- factors based on ALL data will be even larger. H-atoms attached to carbon were placed in calculated positions (C—H = 0.95 - 0.99 Å) and included as riding contributions with isotropic displacement parameters 1.2 - 1.5 times those of the attached atoms.

### (3) rac-6'-Bromo-3'-diethylamino-3H-spiro[2-benzofuran-1,9'-xanthen]-3-one Fractional atomic coordinates and isotropic or equivalent isotropic displacement parameters (Å^2^)

**Table:**

	*x*	*y*	*z*	*U*_iso_*/*U*_eq_	
Br1	0.19033 (4)	0.02441 (3)	0.64991 (3)	0.03278 (14)	
O1	0.1667 (2)	0.41446 (18)	0.49152 (16)	0.0248 (4)	
O2	0.2042 (2)	0.65283 (18)	0.77320 (15)	0.0217 (4)	
O3	0.1991 (3)	0.7974 (2)	0.93699 (17)	0.0301 (4)	
N1	0.2392 (3)	0.7234 (2)	0.25883 (19)	0.0265 (5)	
C1	0.2155 (3)	0.3798 (3)	0.5878 (2)	0.0224 (5)	
C2	0.1813 (3)	0.2475 (3)	0.5752 (2)	0.0257 (5)	
H2	0.1266	0.1871	0.5040	0.031*	
C3	0.2281 (3)	0.2048 (3)	0.6683 (2)	0.0263 (6)	
C4	0.3066 (4)	0.2908 (3)	0.7742 (2)	0.0272 (6)	
H4	0.3360	0.2601	0.8381	0.033*	
C5	0.3404 (3)	0.4226 (3)	0.7836 (2)	0.0256 (5)	
H5	0.3948	0.4826	0.8551	0.031*	
C6	0.2970 (3)	0.4704 (3)	0.6915 (2)	0.0210 (5)	
C7	0.3359 (3)	0.6138 (3)	0.7025 (2)	0.0205 (5)	
C8	0.3204 (3)	0.6420 (3)	0.5850 (2)	0.0213 (5)	
C9	0.3837 (3)	0.7690 (3)	0.5676 (2)	0.0256 (6)	
H9	0.4463	0.8386	0.6313	0.031*	
C10	0.3584 (4)	0.7966 (3)	0.4618 (2)	0.0268 (6)	
H10	0.4044	0.8841	0.4540	0.032*	
C11	0.2647 (3)	0.6962 (3)	0.3641 (2)	0.0228 (5)	
C12	0.1999 (3)	0.5689 (3)	0.3810 (2)	0.0227 (5)	
H12	0.1348	0.4990	0.3182	0.027*	
C13	0.2302 (3)	0.5445 (2)	0.4882 (2)	0.0206 (5)	
C14	0.1323 (3)	0.6234 (3)	0.1601 (2)	0.0265 (6)	
H14A	0.0819	0.6652	0.1101	0.032*	
H14B	0.0366	0.5614	0.1899	0.032*	
C15	0.2312 (4)	0.5483 (3)	0.0865 (3)	0.0342 (7)	
H15A	0.1533	0.4819	0.0223	0.051*	
H15B	0.2806	0.5060	0.1354	0.051*	
H15C	0.3236	0.6087	0.0545	0.051*	
C16	0.3190 (3)	0.8529 (3)	0.2376 (2)	0.0271 (6)	
H16A	0.3361	0.8444	0.1536	0.033*	
H16B	0.4334	0.8949	0.2827	0.033*	
C17	0.2128 (4)	0.9391 (3)	0.2713 (3)	0.0376 (7)	
H17A	0.2746	1.0263	0.2594	0.056*	
H17B	0.1924	0.9457	0.3538	0.056*	
H17C	0.1027	0.9013	0.2227	0.056*	
C18	0.5013 (3)	0.6985 (2)	0.7778 (2)	0.0205 (5)	
C19	0.6676 (3)	0.7027 (3)	0.7611 (3)	0.0267 (6)	
H19	0.6922	0.6516	0.6928	0.032*	
C20	0.7980 (3)	0.7847 (3)	0.8483 (3)	0.0306 (6)	
H20	0.9128	0.7881	0.8399	0.037*	
C21	0.7623 (4)	0.8615 (3)	0.9472 (3)	0.0317 (6)	
H21	0.8530	0.9160	1.0054	0.038*	
C22	0.5968 (4)	0.8599 (3)	0.9619 (2)	0.0270 (6)	
H22	0.5725	0.9142	1.0282	0.032*	
C23	0.4670 (3)	0.7756 (3)	0.8758 (2)	0.0208 (5)	
C24	0.2804 (3)	0.7489 (3)	0.8704 (2)	0.0220 (5)	

### (3) rac-6'-Bromo-3'-diethylamino-3H-spiro[2-benzofuran-1,9'-xanthen]-3-one Atomic displacement parameters (Å^2^)

**Table:**

	*U*^11^	*U*^22^	*U*^33^	*U*^12^	*U*^13^	*U*^23^
Br1	0.02583 (19)	0.02119 (19)	0.0485 (2)	0.00731 (13)	−0.00533 (13)	0.00453 (13)
O1	0.0247 (9)	0.0208 (10)	0.0236 (9)	0.0047 (7)	−0.0068 (7)	0.0006 (7)
O2	0.0143 (8)	0.0264 (10)	0.0211 (8)	0.0080 (7)	−0.0022 (7)	−0.0023 (7)
O3	0.0251 (10)	0.0369 (12)	0.0259 (9)	0.0147 (9)	0.0003 (8)	−0.0039 (8)
N1	0.0280 (12)	0.0273 (12)	0.0217 (11)	0.0089 (10)	−0.0037 (9)	0.0022 (9)
C1	0.0155 (11)	0.0237 (13)	0.0266 (13)	0.0072 (10)	−0.0008 (10)	0.0029 (10)
C2	0.0184 (12)	0.0216 (13)	0.0306 (13)	0.0041 (10)	−0.0037 (10)	−0.0023 (10)
C3	0.0172 (12)	0.0250 (14)	0.0341 (14)	0.0070 (10)	0.0015 (11)	0.0026 (11)
C4	0.0247 (13)	0.0263 (14)	0.0302 (14)	0.0082 (11)	−0.0018 (11)	0.0069 (11)
C5	0.0238 (13)	0.0256 (14)	0.0246 (12)	0.0077 (10)	−0.0021 (10)	0.0015 (10)
C6	0.0176 (11)	0.0195 (13)	0.0243 (12)	0.0066 (9)	0.0003 (10)	0.0017 (9)
C7	0.0155 (11)	0.0222 (13)	0.0220 (12)	0.0072 (9)	−0.0003 (9)	0.0004 (9)
C8	0.0201 (12)	0.0231 (13)	0.0197 (12)	0.0094 (10)	−0.0015 (9)	0.0002 (9)
C9	0.0269 (13)	0.0200 (13)	0.0251 (13)	0.0062 (10)	−0.0049 (10)	−0.0012 (10)
C10	0.0275 (13)	0.0217 (14)	0.0272 (13)	0.0066 (11)	−0.0049 (11)	0.0013 (10)
C11	0.0198 (12)	0.0262 (14)	0.0225 (12)	0.0106 (10)	−0.0022 (10)	0.0019 (10)
C12	0.0196 (12)	0.0249 (14)	0.0213 (12)	0.0094 (10)	−0.0029 (10)	−0.0018 (10)
C13	0.0151 (11)	0.0199 (13)	0.0248 (12)	0.0069 (9)	−0.0019 (9)	−0.0002 (9)
C14	0.0206 (12)	0.0318 (15)	0.0236 (13)	0.0072 (11)	−0.0063 (10)	0.0032 (11)
C15	0.0327 (15)	0.0360 (17)	0.0280 (14)	0.0104 (13)	−0.0036 (12)	−0.0021 (11)
C16	0.0211 (12)	0.0316 (15)	0.0258 (13)	0.0050 (11)	−0.0012 (10)	0.0078 (10)
C17	0.0331 (16)	0.0339 (17)	0.0481 (18)	0.0124 (13)	−0.0026 (13)	0.0138 (13)
C18	0.0162 (11)	0.0200 (12)	0.0239 (12)	0.0050 (9)	−0.0041 (9)	0.0044 (9)
C19	0.0203 (12)	0.0267 (14)	0.0337 (14)	0.0096 (11)	0.0009 (11)	0.0064 (11)
C20	0.0170 (12)	0.0315 (16)	0.0430 (16)	0.0067 (11)	−0.0027 (11)	0.0115 (12)
C21	0.0198 (13)	0.0340 (16)	0.0325 (14)	−0.0010 (11)	−0.0100 (11)	0.0075 (11)
C22	0.0263 (14)	0.0268 (14)	0.0208 (12)	0.0027 (11)	−0.0058 (10)	0.0019 (10)
C23	0.0186 (12)	0.0223 (13)	0.0198 (11)	0.0065 (10)	−0.0019 (9)	0.0029 (9)
C24	0.0197 (12)	0.0256 (14)	0.0186 (12)	0.0082 (10)	−0.0023 (10)	0.0007 (9)

### (3) rac-6'-Bromo-3'-diethylamino-3H-spiro[2-benzofuran-1,9'-xanthen]-3-one Geometric parameters (Å, º)

**Table:**

Br1—C3	1.898 (3)	C11—C12	1.403 (4)
O1—C1	1.369 (3)	C12—C13	1.383 (4)
O1—C13	1.381 (3)	C12—H12	0.9500
O2—C24	1.362 (3)	C14—C15	1.520 (4)
O2—C7	1.510 (3)	C14—H14A	0.9900
O3—C24	1.203 (3)	C14—H14B	0.9900
N1—C11	1.366 (3)	C15—H15A	0.9800
N1—C16	1.455 (4)	C15—H15B	0.9800
N1—C14	1.458 (3)	C15—H15C	0.9800
C1—C2	1.384 (4)	C16—C17	1.512 (4)
C1—C6	1.397 (3)	C16—H16A	0.9900
C2—C3	1.383 (4)	C16—H16B	0.9900
C2—H2	0.9500	C17—H17A	0.9800
C3—C4	1.393 (4)	C17—H17B	0.9800
C4—C5	1.384 (4)	C17—H17C	0.9800
C4—H4	0.9500	C18—C23	1.380 (4)
C5—C6	1.398 (4)	C18—C19	1.386 (4)
C5—H5	0.9500	C19—C20	1.398 (4)
C6—C7	1.502 (4)	C19—H19	0.9500
C7—C8	1.496 (4)	C20—C21	1.390 (5)
C7—C18	1.514 (3)	C20—H20	0.9500
C8—C13	1.391 (3)	C21—C22	1.384 (4)
C8—C9	1.402 (4)	C21—H21	0.9500
C9—C10	1.373 (4)	C22—C23	1.395 (3)
C9—H9	0.9500	C22—H22	0.9500
C10—C11	1.420 (4)	C23—C24	1.475 (3)
C10—H10	0.9500		
			
C1—O1—C13	118.1 (2)	C12—C13—C8	123.2 (2)
C24—O2—C7	111.26 (18)	N1—C14—C15	112.8 (2)
C11—N1—C16	122.3 (2)	N1—C14—H14A	109.0
C11—N1—C14	121.8 (2)	C15—C14—H14A	109.0
C16—N1—C14	115.9 (2)	N1—C14—H14B	109.0
O1—C1—C2	115.3 (2)	C15—C14—H14B	109.0
O1—C1—C6	123.1 (2)	H14A—C14—H14B	107.8
C2—C1—C6	121.6 (2)	C14—C15—H15A	109.5
C3—C2—C1	118.8 (2)	C14—C15—H15B	109.5
C3—C2—H2	120.6	H15A—C15—H15B	109.5
C1—C2—H2	120.6	C14—C15—H15C	109.5
C2—C3—C4	121.9 (3)	H15A—C15—H15C	109.5
C2—C3—Br1	119.1 (2)	H15B—C15—H15C	109.5
C4—C3—Br1	119.0 (2)	N1—C16—C17	112.6 (2)
C5—C4—C3	117.9 (3)	N1—C16—H16A	109.1
C5—C4—H4	121.0	C17—C16—H16A	109.1
C3—C4—H4	121.0	N1—C16—H16B	109.1
C4—C5—C6	122.2 (3)	C17—C16—H16B	109.1
C4—C5—H5	118.9	H16A—C16—H16B	107.8
C6—C5—H5	118.9	C16—C17—H17A	109.5
C1—C6—C5	117.6 (2)	C16—C17—H17B	109.5
C1—C6—C7	120.7 (2)	H17A—C17—H17B	109.5
C5—C6—C7	121.7 (2)	C16—C17—H17C	109.5
C8—C7—C6	110.8 (2)	H17A—C17—H17C	109.5
C8—C7—O2	107.84 (19)	H17B—C17—H17C	109.5
C6—C7—O2	107.9 (2)	C23—C18—C19	120.9 (2)
C8—C7—C18	114.7 (2)	C23—C18—C7	110.1 (2)
C6—C7—C18	113.2 (2)	C19—C18—C7	129.0 (2)
O2—C7—C18	101.64 (19)	C18—C19—C20	117.7 (3)
C13—C8—C9	115.9 (2)	C18—C19—H19	121.2
C13—C8—C7	121.6 (2)	C20—C19—H19	121.2
C9—C8—C7	122.3 (2)	C21—C20—C19	121.1 (3)
C10—C9—C8	122.5 (2)	C21—C20—H20	119.4
C10—C9—H9	118.8	C19—C20—H20	119.4
C8—C9—H9	118.8	C22—C21—C20	121.0 (2)
C9—C10—C11	121.0 (3)	C22—C21—H21	119.5
C9—C10—H10	119.5	C20—C21—H21	119.5
C11—C10—H10	119.5	C21—C22—C23	117.5 (3)
N1—C11—C12	122.0 (2)	C21—C22—H22	121.3
N1—C11—C10	121.1 (2)	C23—C22—H22	121.3
C12—C11—C10	116.9 (2)	C18—C23—C22	121.8 (2)
C13—C12—C11	120.5 (2)	C18—C23—C24	108.6 (2)
C13—C12—H12	119.7	C22—C23—C24	129.6 (2)
C11—C12—H12	119.7	O3—C24—O2	122.2 (2)
O1—C13—C12	114.7 (2)	O3—C24—C23	129.5 (2)
O1—C13—C8	122.1 (2)	O2—C24—C23	108.3 (2)
			
C13—O1—C1—C2	−165.8 (2)	C9—C10—C11—C12	0.0 (4)
C13—O1—C1—C6	13.1 (4)	N1—C11—C12—C13	179.1 (2)
O1—C1—C2—C3	179.4 (2)	C10—C11—C12—C13	−1.0 (4)
C6—C1—C2—C3	0.5 (4)	C1—O1—C13—C12	169.3 (2)
C1—C2—C3—C4	0.8 (4)	C1—O1—C13—C8	−9.3 (3)
C1—C2—C3—Br1	−177.47 (19)	C11—C12—C13—O1	−176.9 (2)
C2—C3—C4—C5	−1.3 (4)	C11—C12—C13—C8	1.7 (4)
Br1—C3—C4—C5	176.9 (2)	C9—C8—C13—O1	177.3 (2)
C3—C4—C5—C6	0.6 (4)	C7—C8—C13—O1	−7.6 (4)
O1—C1—C6—C5	−180.0 (2)	C9—C8—C13—C12	−1.2 (4)
C2—C1—C6—C5	−1.2 (4)	C7—C8—C13—C12	173.9 (2)
O1—C1—C6—C7	0.1 (4)	C11—N1—C14—C15	−87.3 (3)
C2—C1—C6—C7	178.9 (2)	C16—N1—C14—C15	91.6 (3)
C4—C5—C6—C1	0.6 (4)	C11—N1—C16—C17	−87.7 (3)
C4—C5—C6—C7	−179.4 (2)	C14—N1—C16—C17	93.4 (3)
C1—C6—C7—C8	−15.1 (3)	C8—C7—C18—C23	115.2 (2)
C5—C6—C7—C8	164.9 (2)	C6—C7—C18—C23	−116.3 (2)
C1—C6—C7—O2	102.7 (3)	O2—C7—C18—C23	−0.9 (3)
C5—C6—C7—O2	−77.3 (3)	C8—C7—C18—C19	−66.8 (4)
C1—C6—C7—C18	−145.7 (2)	C6—C7—C18—C19	61.7 (4)
C5—C6—C7—C18	34.4 (3)	O2—C7—C18—C19	177.1 (3)
C24—O2—C7—C8	−118.3 (2)	C23—C18—C19—C20	1.8 (4)
C24—O2—C7—C6	122.0 (2)	C7—C18—C19—C20	−176.0 (3)
C24—O2—C7—C18	2.7 (3)	C18—C19—C20—C21	−1.4 (4)
C6—C7—C8—C13	18.8 (3)	C19—C20—C21—C22	−0.5 (5)
O2—C7—C8—C13	−99.0 (3)	C20—C21—C22—C23	1.9 (4)
C18—C7—C8—C13	148.5 (2)	C19—C18—C23—C22	−0.4 (4)
C6—C7—C8—C9	−166.4 (2)	C7—C18—C23—C22	177.8 (2)
O2—C7—C8—C9	75.8 (3)	C19—C18—C23—C24	−179.2 (2)
C18—C7—C8—C9	−36.7 (3)	C7—C18—C23—C24	−1.0 (3)
C13—C8—C9—C10	0.1 (4)	C21—C22—C23—C18	−1.5 (4)
C7—C8—C9—C10	−174.9 (3)	C21—C22—C23—C24	177.1 (3)
C8—C9—C10—C11	0.5 (4)	C7—O2—C24—O3	177.5 (2)
C16—N1—C11—C12	−174.2 (2)	C7—O2—C24—C23	−3.4 (3)
C14—N1—C11—C12	4.7 (4)	C18—C23—C24—O3	−178.2 (3)
C16—N1—C11—C10	5.9 (4)	C22—C23—C24—O3	3.1 (5)
C14—N1—C11—C10	−175.2 (2)	C18—C23—C24—O2	2.7 (3)
C9—C10—C11—N1	179.9 (3)	C22—C23—C24—O2	−175.9 (3)

### (3) rac-6'-Bromo-3'-diethylamino-3H-spiro[2-benzofuran-1,9'-xanthen]-3-one Hydrogen-bond geometry (Å, º)

Cg is the centroid of the C8–C13 ring.

**Table:**

*D*—H···*A*	*D*—H	H···*A*	*D*···*A*	*D*—H···*A*
C14—H14*B*···O2^i^	0.99	2.68	3.649 (4)	165
C16—H16*A*···O3^ii^	0.99	2.64	3.522 (3)	148
C16—H16*B*···Br1^iii^	0.99	2.99	3.939 (3)	162
C17—H17*A*···*Cg*^iv^	0.98	2.75	3.666 (4)	156
C19—H19···O1^iii^	0.95	2.57	3.485 (4)	161
C20—H20···O3^v^	0.95	2.58	3.421 (3)	148

Symmetry codes: (i) −*x*, −*y*+1, −*z*+1; (ii) *x*, *y*, *z*−1; (iii) −*x*+1, −*y*+1, −*z*+1; (iv) −*x*+1, −*y*+2, −*z*+1; (v) *x*+1, *y*, *z*.

### (4) (1S)-6'-Bromo-3'-diethylamino-3H-spiro[2-benzofuran-1,9'-xanthen]-3-one Crystal data

**Table:**

C_24_H_20_BrNO_3_	*D*_x_ = 1.488 Mg m^−^^3^
*M**_r_* = 450.32	Mo *K*α radiation, λ = 0.71073 Å
Orthorhombic, *P*2_1_2_1_2_1_	Cell parameters from 9874 reflections
*a* = 11.0772 (6) Å	θ = 2.4–28.7°
*b* = 13.0582 (8) Å	µ = 2.07 mm^−^^1^
*c* = 13.8966 (8) Å	*T* = 100 K
*V* = 2010.1 (2) Å^3^	Column, colourless
*Z* = 4	0.31 × 0.12 × 0.10 mm
*F*(000) = 920	

### (4) (1S)-6'-Bromo-3'-diethylamino-3H-spiro[2-benzofuran-1,9'-xanthen]-3-one Data collection

**Table:**

Bruker SMART APEX CCD diffractometer	5417 independent reflections
Radiation source: fine-focus sealed tube	4926 reflections with *I* > 2σ(*I*)
Graphite monochromator	*R*_int_ = 0.043
Detector resolution: 8.3333 pixels mm^-1^	θ_max_ = 29.2°, θ_min_ = 2.1°
φ and ω scans	*h* = −15→15
Absorption correction: multi-scan (*SADABS*; Bruker, 2016)	*k* = −17→17
*T*_min_ = 0.69, *T*_max_ = 0.82	*l* = −18→18
39006 measured reflections	

### (4) (1S)-6'-Bromo-3'-diethylamino-3H-spiro[2-benzofuran-1,9'-xanthen]-3-one Refinement

**Table:**

Refinement on *F*^2^	Secondary atom site location: difference Fourier map
Least-squares matrix: full	Hydrogen site location: mixed
*R*[*F*^2^ > 2σ(*F*^2^)] = 0.026	H atoms treated by a mixture of independent and constrained refinement
*wR*(*F*^2^) = 0.058	*w* = 1/[σ^2^(*F*_o_^2^) + (0.0299*P*)^2^] where *P* = (*F*_o_^2^ + 2*F*_c_^2^)/3
*S* = 0.99	(Δ/σ)_max_ = 0.001
5417 reflections	Δρ_max_ = 0.62 e Å^−^^3^
320 parameters	Δρ_min_ = −0.25 e Å^−^^3^
0 restraints	Absolute structure: Flack *x* determined using 1981 quotients [(*I*^+^)-(*I*^-^)]/[(*I*^+^)+(*I*^-^) (Parsons *et al.*, 2013)
Primary atom site location: structure-invariant direct methods	Absolute structure parameter: −0.006 (3)

### (4) (1S)-6'-Bromo-3'-diethylamino-3H-spiro[2-benzofuran-1,9'-xanthen]-3-one Special details

**Table:**

Experimental. The diffraction data were obtained from 3 sets of 400 frames, each of width 0.5° in ω, colllected at φ = 0.00, 90.00 and 180.00° and 2 sets of 800 frames, each of width 0.45° in φ, collected at ω = –30.00 and 210.00°. The scan time was 20 sec/frame.
Geometry. All esds (except the esd in the dihedral angle between two l.s. planes) are estimated using the full covariance matrix. The cell esds are taken into account individually in the estimation of esds in distances, angles and torsion angles; correlations between esds in cell parameters are only used when they are defined by crystal symmetry. An approximate (isotropic) treatment of cell esds is used for estimating esds involving l.s. planes.
Refinement. Refinement of F^2^ against ALL reflections. The weighted R-factor wR and goodness of fit S are based on F^2^, conventional R-factors R are based on F, with F set to zero for negative F^2^. The threshold expression of F^2^ > 2sigma(F^2^) is used only for calculating R-factors(gt) etc. and is not relevant to the choice of reflections for refinement. R-factors based on F^2^ are statistically about twice as large as those based on F, and R- factors based on ALL data will be even larger.

### (4) (1S)-6'-Bromo-3'-diethylamino-3H-spiro[2-benzofuran-1,9'-xanthen]-3-one Fractional atomic coordinates and isotropic or equivalent isotropic displacement parameters (Å^2^)

**Table:**

	*x*	*y*	*z*	*U*_iso_*/*U*_eq_	
Br1	0.23778 (2)	0.57329 (2)	0.58190 (2)	0.02156 (7)	
O1	0.62242 (14)	0.63049 (13)	0.37859 (12)	0.0174 (4)	
O2	0.83447 (14)	0.44945 (12)	0.52206 (11)	0.0149 (3)	
O3	0.96324 (16)	0.34761 (13)	0.60162 (12)	0.0232 (4)	
N1	0.93770 (19)	0.75727 (16)	0.17844 (14)	0.0191 (4)	
C1	0.5808 (2)	0.59393 (17)	0.46462 (16)	0.0132 (5)	
C2	0.4557 (2)	0.59644 (18)	0.47625 (18)	0.0166 (5)	
H2	0.412 (3)	0.617 (2)	0.427 (2)	0.023 (7)*	
C3	0.40723 (19)	0.56383 (17)	0.56230 (16)	0.0154 (5)	
C4	0.4786 (2)	0.52661 (19)	0.63625 (18)	0.0178 (5)	
H4	0.440 (2)	0.5053 (19)	0.693 (2)	0.017 (7)*	
C5	0.6020 (2)	0.52317 (19)	0.62259 (18)	0.0163 (5)	
H5	0.651 (3)	0.495 (2)	0.6671 (19)	0.020 (8)*	
C6	0.6556 (2)	0.55733 (18)	0.53668 (17)	0.0138 (5)	
C7	0.79072 (19)	0.55778 (16)	0.52627 (16)	0.0126 (4)	
C8	0.8285 (2)	0.61105 (17)	0.43527 (16)	0.0132 (4)	
C9	0.9502 (2)	0.63061 (18)	0.41480 (19)	0.0170 (5)	
H9	1.007 (2)	0.6057 (19)	0.4633 (18)	0.012 (6)*	
C10	0.9872 (2)	0.67728 (19)	0.33110 (18)	0.0178 (5)	
H10	1.070 (3)	0.685 (2)	0.3211 (19)	0.020 (7)*	
C11	0.9017 (2)	0.70912 (17)	0.26139 (16)	0.0148 (5)	
C12	0.7793 (2)	0.68911 (17)	0.28086 (17)	0.0152 (5)	
H12	0.722 (2)	0.7083 (19)	0.2415 (17)	0.013 (6)*	
C13	0.7462 (2)	0.64179 (15)	0.36616 (15)	0.0129 (4)	
C14	0.8504 (2)	0.7873 (2)	0.10481 (18)	0.0227 (5)	
H14A	0.787 (3)	0.733 (2)	0.098 (2)	0.031 (8)*	
H14B	0.891 (2)	0.789 (2)	0.0417 (18)	0.014 (6)*	
C15	0.7924 (2)	0.8904 (2)	0.1260 (2)	0.0285 (6)	
H15A	0.7563	0.8890	0.1903	0.043*	
H15B	0.7296	0.9043	0.0781	0.043*	
H15C	0.8539	0.9442	0.1231	0.043*	
C16	1.0602 (2)	0.7975 (2)	0.16496 (19)	0.0198 (5)	
H16A	1.047 (2)	0.866 (2)	0.1302 (18)	0.017 (7)*	
H16B	1.089 (2)	0.820 (2)	0.230 (2)	0.021 (7)*	
C17	1.1432 (2)	0.7252 (2)	0.1109 (2)	0.0253 (6)	
H17A	1.1564	0.6633	0.1493	0.038*	
H17B	1.2208	0.7591	0.0990	0.038*	
H17C	1.1062	0.7066	0.0493	0.038*	
C18	0.8524 (2)	0.59745 (18)	0.61583 (17)	0.0139 (5)	
C19	0.8402 (2)	0.69192 (19)	0.65983 (18)	0.0179 (5)	
H19	0.788 (2)	0.739 (2)	0.6336 (19)	0.019 (7)*	
C20	0.9055 (2)	0.7091 (2)	0.74369 (19)	0.0228 (5)	
H20	0.909 (3)	0.770 (3)	0.779 (2)	0.039 (9)*	
C21	0.9793 (2)	0.6332 (2)	0.78279 (19)	0.0230 (6)	
H21	1.024 (2)	0.649 (2)	0.8452 (19)	0.021 (7)*	
C22	0.9906 (2)	0.5390 (2)	0.73893 (18)	0.0202 (5)	
H22	1.040 (2)	0.4897 (19)	0.7606 (19)	0.013 (7)*	
C23	0.9256 (2)	0.52284 (19)	0.65427 (18)	0.0158 (5)	
C24	0.91472 (18)	0.42975 (18)	0.59411 (15)	0.0151 (4)	

### (4) (1S)-6'-Bromo-3'-diethylamino-3H-spiro[2-benzofuran-1,9'-xanthen]-3-one Atomic displacement parameters (Å^2^)

**Table:**

	*U*^11^	*U*^22^	*U*^33^	*U*^12^	*U*^13^	*U*^23^
Br1	0.01162 (10)	0.03082 (12)	0.02224 (12)	−0.00078 (9)	0.00204 (9)	−0.00572 (10)
O1	0.0111 (8)	0.0258 (9)	0.0152 (8)	−0.0016 (7)	−0.0013 (7)	0.0059 (7)
O2	0.0166 (8)	0.0134 (8)	0.0148 (8)	0.0025 (6)	−0.0012 (6)	0.0002 (6)
O3	0.0250 (9)	0.0233 (9)	0.0215 (10)	0.0091 (7)	−0.0017 (8)	0.0044 (7)
N1	0.0158 (10)	0.0272 (11)	0.0142 (10)	−0.0032 (9)	0.0012 (8)	0.0036 (9)
C1	0.0139 (11)	0.0135 (11)	0.0123 (11)	−0.0014 (8)	0.0008 (9)	−0.0005 (8)
C2	0.0135 (11)	0.0208 (12)	0.0155 (12)	0.0006 (9)	−0.0034 (9)	0.0008 (9)
C3	0.0106 (9)	0.0156 (10)	0.0199 (12)	−0.0024 (9)	0.0005 (8)	−0.0047 (10)
C4	0.0176 (12)	0.0199 (12)	0.0161 (12)	−0.0039 (9)	0.0022 (10)	0.0019 (10)
C5	0.0159 (12)	0.0192 (11)	0.0138 (12)	−0.0002 (9)	−0.0023 (9)	0.0025 (10)
C6	0.0121 (10)	0.0130 (11)	0.0162 (11)	0.0002 (9)	0.0005 (9)	0.0008 (9)
C7	0.0127 (10)	0.0124 (10)	0.0127 (10)	0.0014 (8)	−0.0002 (8)	0.0001 (8)
C8	0.0125 (10)	0.0159 (10)	0.0111 (11)	0.0020 (8)	0.0004 (8)	0.0009 (9)
C9	0.0132 (10)	0.0221 (11)	0.0157 (11)	0.0019 (9)	−0.0022 (11)	0.0012 (11)
C10	0.0119 (11)	0.0243 (13)	0.0173 (12)	−0.0011 (10)	0.0021 (9)	0.0009 (10)
C11	0.0176 (11)	0.0146 (10)	0.0123 (11)	0.0002 (9)	0.0021 (9)	−0.0019 (9)
C12	0.0148 (11)	0.0179 (11)	0.0130 (11)	0.0005 (9)	−0.0024 (9)	0.0006 (8)
C13	0.0099 (10)	0.0143 (9)	0.0146 (10)	0.0000 (9)	−0.0001 (9)	−0.0019 (8)
C14	0.0230 (13)	0.0319 (14)	0.0133 (12)	−0.0058 (11)	−0.0017 (10)	0.0062 (10)
C15	0.0240 (14)	0.0340 (14)	0.0275 (14)	−0.0012 (11)	−0.0029 (11)	0.0130 (12)
C16	0.0191 (12)	0.0241 (13)	0.0162 (12)	−0.0046 (10)	0.0026 (10)	0.0020 (10)
C17	0.0207 (12)	0.0305 (14)	0.0248 (14)	−0.0004 (11)	0.0038 (10)	−0.0014 (11)
C18	0.0092 (10)	0.0201 (11)	0.0124 (10)	−0.0034 (8)	0.0010 (8)	0.0010 (9)
C19	0.0150 (12)	0.0175 (12)	0.0210 (13)	−0.0009 (9)	0.0006 (10)	−0.0012 (10)
C20	0.0182 (12)	0.0271 (14)	0.0231 (14)	−0.0071 (11)	0.0028 (10)	−0.0078 (11)
C21	0.0164 (12)	0.0373 (15)	0.0152 (12)	−0.0080 (11)	−0.0015 (10)	−0.0020 (11)
C22	0.0128 (11)	0.0318 (14)	0.0159 (12)	−0.0015 (10)	−0.0009 (10)	0.0062 (10)
C23	0.0116 (11)	0.0210 (12)	0.0149 (12)	−0.0004 (9)	0.0017 (9)	0.0033 (10)
C24	0.0120 (9)	0.0199 (10)	0.0133 (11)	0.0011 (9)	0.0024 (8)	0.0033 (10)

### (4) (1S)-6'-Bromo-3'-diethylamino-3H-spiro[2-benzofuran-1,9'-xanthen]-3-one Geometric parameters (Å, º)

**Table:**

Br1—C3	1.901 (2)	C11—C12	1.408 (3)
O1—C1	1.367 (3)	C12—C13	1.386 (3)
O1—C13	1.389 (3)	C12—H12	0.87 (2)
O2—C24	1.363 (3)	C14—C15	1.520 (4)
O2—C7	1.496 (3)	C14—H14A	1.01 (3)
O3—C24	1.204 (3)	C14—H14B	0.98 (3)
N1—C11	1.372 (3)	C15—H15A	0.9800
N1—C14	1.461 (3)	C15—H15B	0.9800
N1—C16	1.467 (3)	C15—H15C	0.9800
C1—C6	1.385 (3)	C16—C17	1.517 (4)
C1—C2	1.395 (3)	C16—H16A	1.02 (3)
C2—C3	1.378 (3)	C16—H16B	0.99 (3)
C2—H2	0.88 (3)	C17—H17A	0.9800
C3—C4	1.385 (3)	C17—H17B	0.9800
C4—C5	1.381 (4)	C17—H17C	0.9800
C4—H4	0.93 (3)	C18—C23	1.375 (3)
C5—C6	1.406 (3)	C18—C19	1.384 (3)
C5—H5	0.90 (3)	C19—C20	1.390 (3)
C6—C7	1.504 (3)	C19—H19	0.92 (3)
C7—C8	1.503 (3)	C20—C21	1.395 (4)
C7—C18	1.512 (3)	C20—H20	0.93 (3)
C8—C13	1.384 (3)	C21—C22	1.378 (4)
C8—C9	1.401 (3)	C21—H21	1.02 (3)
C9—C10	1.376 (3)	C22—C23	1.396 (3)
C9—H9	0.98 (3)	C22—H22	0.90 (3)
C10—C11	1.417 (3)	C23—C24	1.480 (3)
C10—H10	0.93 (3)		
			
C1—O1—C13	118.57 (17)	C12—C13—O1	114.49 (19)
C24—O2—C7	111.15 (17)	N1—C14—C15	112.5 (2)
C11—N1—C14	121.3 (2)	N1—C14—H14A	109.6 (17)
C11—N1—C16	122.7 (2)	C15—C14—H14A	110.3 (16)
C14—N1—C16	115.2 (2)	N1—C14—H14B	109.3 (15)
O1—C1—C6	123.4 (2)	C15—C14—H14B	110.3 (15)
O1—C1—C2	115.3 (2)	H14A—C14—H14B	105 (2)
C6—C1—C2	121.2 (2)	C14—C15—H15A	109.5
C3—C2—C1	118.7 (2)	C14—C15—H15B	109.5
C3—C2—H2	123.4 (18)	H15A—C15—H15B	109.5
C1—C2—H2	117.9 (18)	C14—C15—H15C	109.5
C2—C3—C4	122.0 (2)	H15A—C15—H15C	109.5
C2—C3—Br1	119.29 (17)	H15B—C15—H15C	109.5
C4—C3—Br1	118.69 (17)	N1—C16—C17	113.6 (2)
C5—C4—C3	118.3 (2)	N1—C16—H16A	104.1 (15)
C5—C4—H4	123.7 (17)	C17—C16—H16A	113.0 (15)
C3—C4—H4	118.0 (17)	N1—C16—H16B	106.5 (16)
C4—C5—C6	121.6 (2)	C17—C16—H16B	115.7 (16)
C4—C5—H5	120.9 (19)	H16A—C16—H16B	103 (2)
C6—C5—H5	117.3 (19)	C16—C17—H17A	109.5
C1—C6—C5	118.1 (2)	C16—C17—H17B	109.5
C1—C6—C7	121.6 (2)	H17A—C17—H17B	109.5
C5—C6—C7	120.2 (2)	C16—C17—H17C	109.5
O2—C7—C8	108.32 (17)	H17A—C17—H17C	109.5
O2—C7—C6	108.81 (18)	H17B—C17—H17C	109.5
C8—C7—C6	111.12 (19)	C23—C18—C19	121.2 (2)
O2—C7—C18	102.10 (17)	C23—C18—C7	110.1 (2)
C8—C7—C18	114.09 (18)	C19—C18—C7	128.7 (2)
C6—C7—C18	111.86 (19)	C18—C19—C20	117.6 (2)
C13—C8—C9	116.1 (2)	C18—C19—H19	119.1 (17)
C13—C8—C7	122.29 (19)	C20—C19—H19	123.2 (17)
C9—C8—C7	121.6 (2)	C19—C20—C21	121.1 (3)
C10—C9—C8	122.6 (2)	C19—C20—H20	127 (2)
C10—C9—H9	122.6 (15)	C21—C20—H20	112 (2)
C8—C9—H9	114.8 (15)	C22—C21—C20	121.0 (2)
C9—C10—C11	120.6 (2)	C22—C21—H21	120.7 (16)
C9—C10—H10	118.0 (17)	C20—C21—H21	118.3 (16)
C11—C10—H10	121.4 (17)	C21—C22—C23	117.5 (2)
N1—C11—C12	121.8 (2)	C21—C22—H22	123.2 (16)
N1—C11—C10	121.0 (2)	C23—C22—H22	119.3 (16)
C12—C11—C10	117.3 (2)	C18—C23—C22	121.6 (2)
C13—C12—C11	120.1 (2)	C18—C23—C24	108.3 (2)
C13—C12—H12	118.2 (16)	C22—C23—C24	130.0 (2)
C11—C12—H12	121.6 (16)	O3—C24—O2	121.5 (2)
C8—C13—C12	123.2 (2)	O3—C24—C23	130.2 (2)
C8—C13—O1	122.23 (18)	O2—C24—C23	108.23 (19)
			
C13—O1—C1—C6	−7.0 (3)	C9—C10—C11—C12	−1.2 (3)
C13—O1—C1—C2	172.46 (19)	N1—C11—C12—C13	−178.9 (2)
O1—C1—C2—C3	−178.0 (2)	C10—C11—C12—C13	1.2 (3)
C6—C1—C2—C3	1.5 (3)	C9—C8—C13—C12	0.2 (3)
C1—C2—C3—C4	−1.5 (3)	C7—C8—C13—C12	−178.6 (2)
C1—C2—C3—Br1	176.79 (17)	C9—C8—C13—O1	−177.6 (2)
C2—C3—C4—C5	0.5 (4)	C7—C8—C13—O1	3.6 (3)
Br1—C3—C4—C5	−177.88 (18)	C11—C12—C13—C8	−0.7 (3)
C3—C4—C5—C6	0.7 (4)	C11—C12—C13—O1	177.3 (2)
O1—C1—C6—C5	179.1 (2)	C1—O1—C13—C8	4.4 (3)
C2—C1—C6—C5	−0.4 (3)	C1—O1—C13—C12	−173.62 (18)
O1—C1—C6—C7	1.7 (3)	C11—N1—C14—C15	84.3 (3)
C2—C1—C6—C7	−177.8 (2)	C16—N1—C14—C15	−85.6 (3)
C4—C5—C6—C1	−0.7 (4)	C11—N1—C16—C17	96.7 (3)
C4—C5—C6—C7	176.7 (2)	C14—N1—C16—C17	−93.5 (3)
C24—O2—C7—C8	117.59 (19)	O2—C7—C18—C23	2.5 (2)
C24—O2—C7—C6	−121.50 (19)	C8—C7—C18—C23	−114.1 (2)
C24—O2—C7—C18	−3.1 (2)	C6—C7—C18—C23	118.7 (2)
C1—C6—C7—O2	−113.6 (2)	O2—C7—C18—C19	−175.5 (2)
C5—C6—C7—O2	69.1 (3)	C8—C7—C18—C19	67.9 (3)
C1—C6—C7—C8	5.6 (3)	C6—C7—C18—C19	−59.3 (3)
C5—C6—C7—C8	−171.8 (2)	C23—C18—C19—C20	0.7 (3)
C1—C6—C7—C18	134.4 (2)	C7—C18—C19—C20	178.5 (2)
C5—C6—C7—C18	−43.0 (3)	C18—C19—C20—C21	−1.0 (4)
O2—C7—C8—C13	111.4 (2)	C19—C20—C21—C22	0.6 (4)
C6—C7—C8—C13	−8.1 (3)	C20—C21—C22—C23	0.0 (4)
C18—C7—C8—C13	−135.7 (2)	C19—C18—C23—C22	−0.1 (4)
O2—C7—C8—C9	−67.3 (3)	C7—C18—C23—C22	−178.3 (2)
C6—C7—C8—C9	173.2 (2)	C19—C18—C23—C24	177.1 (2)
C18—C7—C8—C9	45.6 (3)	C7—C18—C23—C24	−1.1 (3)
C13—C8—C9—C10	−0.2 (3)	C21—C22—C23—C18	−0.3 (4)
C7—C8—C9—C10	178.6 (2)	C21—C22—C23—C24	−176.8 (2)
C8—C9—C10—C11	0.8 (4)	C7—O2—C24—O3	−177.8 (2)
C14—N1—C11—C12	−1.9 (3)	C7—O2—C24—C23	2.6 (2)
C16—N1—C11—C12	167.3 (2)	C18—C23—C24—O3	179.5 (2)
C14—N1—C11—C10	178.0 (2)	C22—C23—C24—O3	−3.6 (4)
C16—N1—C11—C10	−12.9 (4)	C18—C23—C24—O2	−0.9 (3)
C9—C10—C11—N1	178.9 (2)	C22—C23—C24—O2	176.0 (2)

### (4) (1S)-6'-Bromo-3'-diethylamino-3H-spiro[2-benzofuran-1,9'-xanthen]-3-one Hydrogen-bond geometry (Å, º)

**Table:**

*D*—H···*A*	*D*—H	H···*A*	*D*···*A*	*D*—H···*A*
C14—H14*B*···O3^i^	0.99 (2)	2.68 (2)	3.621 (3)	160.7 (19)
C20—H20···O3^ii^	0.94 (2)	2.41 (3)	3.163 (3)	138 (3)

Symmetry codes: (i) −*x*+2, *y*+1/2, −*z*+1/2; (ii) −*x*+2, *y*+1/2, −*z*+3/2.

### (5) (1R)-6'-Bromo-3'-diethylamino-3H-spiro[2-benzofuran-1,9'-xanthen]-3-one Crystal data

**Table:**

C_24_H_20_BrNO_3_	*D*_x_ = 1.502 Mg m^−^^3^
*M**_r_* = 450.32	Mo *K*α radiation, λ = 0.71073 Å
Orthorhombic, *P*2_1_2_1_2_1_	Cell parameters from 7498 reflections
*a* = 8.1529 (13) Å	θ = 2.2–25.3°
*b* = 18.185 (3) Å	µ = 2.09 mm^−^^1^
*c* = 26.860 (4) Å	*T* = 100 K
*V* = 3982.3 (11) Å^3^	Column, colourless
*Z* = 8	0.26 × 0.06 × 0.04 mm
*F*(000) = 1840	

### (5) (1R)-6'-Bromo-3'-diethylamino-3H-spiro[2-benzofuran-1,9'-xanthen]-3-one Data collection

**Table:**

Bruker SMART APEX CCD diffractometer	10174 independent reflections
Radiation source: fine-focus sealed tube	7285 reflections with *I* > 2σ(*I*)
Graphite monochromator	*R*_int_ = 0.075
Detector resolution: 8.3333 pixels mm^-1^	θ_max_ = 29.1°, θ_min_ = 1.4°
φ and ω scans	*h* = −10→10
Absorption correction: multi-scan (*SADABS*; Bruker, 2016)	*k* = −23→24
*T*_min_ = 0.70, *T*_max_ = 0.92	*l* = −35→36
38240 measured reflections	

### (5) (1R)-6'-Bromo-3'-diethylamino-3H-spiro[2-benzofuran-1,9'-xanthen]-3-one Refinement

**Table:**

Refinement on *F*^2^	Secondary atom site location: difference Fourier map
Least-squares matrix: full	Hydrogen site location: inferred from neighbouring sites
*R*[*F*^2^ > 2σ(*F*^2^)] = 0.045	H-atom parameters constrained
*wR*(*F*^2^) = 0.093	*w* = 1/[σ^2^(*F*_o_^2^) + (0.0089*P*)^2^] where *P* = (*F*_o_^2^ + 2*F*_c_^2^)/3
*S* = 0.97	(Δ/σ)_max_ = 0.001
10174 reflections	Δρ_max_ = 0.92 e Å^−^^3^
527 parameters	Δρ_min_ = −0.34 e Å^−^^3^
0 restraints	Absolute structure: Flack *x* determined using 2575 quotients [(*I*^+^)-(*I*^-^)]/[(*I*^+^)+(*I*^-^) (Parsons *et al.*, 2013)
Primary atom site location: structure-invariant direct methods	Absolute structure parameter: −0.002 (6)

### (5) (1R)-6'-Bromo-3'-diethylamino-3H-spiro[2-benzofuran-1,9'-xanthen]-3-one Special details

**Table:**

Experimental. The diffraction data were collected in three sets of 363 frames (0.5° width in ω) at φ = 0, 120 and 240°. A scan time of 60 sec/frame was used.
Geometry. All esds (except the esd in the dihedral angle between two l.s. planes) are estimated using the full covariance matrix. The cell esds are taken into account individually in the estimation of esds in distances, angles and torsion angles; correlations between esds in cell parameters are only used when they are defined by crystal symmetry. An approximate (isotropic) treatment of cell esds is used for estimating esds involving l.s. planes.
Refinement. Refinement of F^2^ against ALL reflections. The weighted R-factor wR and goodness of fit S are based on F^2^, conventional R-factors R are based on F, with F set to zero for negative F^2^. The threshold expression of F^2^ > 2sigma(F^2^) is used only for calculating R-factors(gt) etc. and is not relevant to the choice of reflections for refinement. R-factors based on F^2^ are statistically about twice as large as those based on F, and R- factors based on ALL data will be even larger. H-atoms attached to carbon were placed in calculated positions (C—H = 0.95 - 0.99 Å). All were included as riding contributions with isotropic displacement parameters 1.2 - 1.5 times those of the attached atoms.

### (5) (1R)-6'-Bromo-3'-diethylamino-3H-spiro[2-benzofuran-1,9'-xanthen]-3-one Fractional atomic coordinates and isotropic or equivalent isotropic displacement parameters (Å^2^)

**Table:**

	*x*	*y*	*z*	*U*_iso_*/*U*_eq_	
Br1	0.52585 (6)	0.63496 (3)	0.28218 (2)	0.02849 (13)	
O1	0.6662 (4)	0.57150 (16)	0.46407 (11)	0.0189 (7)	
O2	0.4245 (3)	0.39341 (16)	0.46124 (11)	0.0153 (7)	
O3	0.2630 (4)	0.29559 (18)	0.44638 (11)	0.0207 (7)	
N1	0.8582 (5)	0.5506 (2)	0.63051 (13)	0.0178 (8)	
C1	0.6260 (5)	0.5413 (2)	0.41884 (16)	0.0160 (10)	
C2	0.6056 (5)	0.5918 (2)	0.38032 (16)	0.0181 (10)	
H2	0.6212	0.6429	0.3860	0.022*	
C3	0.5626 (5)	0.5663 (3)	0.33398 (16)	0.0191 (10)	
C4	0.5443 (5)	0.4916 (3)	0.32441 (16)	0.0199 (10)	
H4	0.5175	0.4747	0.2920	0.024*	
C5	0.5659 (5)	0.4425 (2)	0.36318 (16)	0.0180 (10)	
H5	0.5552	0.3913	0.3571	0.022*	
C6	0.6035 (5)	0.4671 (2)	0.41148 (16)	0.0149 (9)	
C7	0.6029 (5)	0.4136 (2)	0.45477 (15)	0.0136 (9)	
C8	0.6652 (5)	0.4501 (2)	0.50108 (15)	0.0130 (9)	
C9	0.6964 (5)	0.4098 (2)	0.54461 (16)	0.0157 (10)	
H9	0.6778	0.3583	0.5443	0.019*	
C10	0.7528 (5)	0.4418 (2)	0.58754 (16)	0.0137 (9)	
H10	0.7699	0.4124	0.6163	0.016*	
C11	0.7859 (5)	0.5186 (2)	0.58949 (16)	0.0152 (9)	
C12	0.7507 (5)	0.5598 (2)	0.54662 (16)	0.0156 (10)	
H12	0.7657	0.6116	0.5467	0.019*	
C13	0.6939 (5)	0.5246 (2)	0.50400 (16)	0.0147 (9)	
C14	0.8803 (6)	0.5091 (3)	0.67630 (16)	0.0214 (11)	
H14A	0.9644	0.5337	0.6970	0.026*	
H14B	0.9215	0.4594	0.6680	0.026*	
C15	0.7228 (6)	0.5018 (3)	0.70632 (17)	0.0221 (11)	
H15A	0.6887	0.5504	0.7182	0.033*	
H15B	0.7420	0.4693	0.7349	0.033*	
H15C	0.6365	0.4809	0.6852	0.033*	
C16	0.8935 (5)	0.6297 (3)	0.63138 (17)	0.0219 (10)	
H16A	0.9442	0.6437	0.5993	0.026*	
H16B	0.9746	0.6396	0.6580	0.026*	
C17	0.7432 (6)	0.6778 (3)	0.64011 (19)	0.0303 (13)	
H17A	0.7756	0.7297	0.6393	0.045*	
H17B	0.6955	0.6663	0.6727	0.045*	
H17C	0.6620	0.6684	0.6140	0.045*	
C18	0.6792 (5)	0.3401 (2)	0.44466 (15)	0.0131 (9)	
C19	0.8454 (5)	0.3221 (2)	0.44054 (16)	0.0153 (10)	
H19	0.9282	0.3586	0.4430	0.018*	
C20	0.8845 (6)	0.2494 (2)	0.43276 (15)	0.0179 (10)	
H20	0.9964	0.2357	0.4295	0.022*	
C21	0.7630 (6)	0.1948 (2)	0.42956 (16)	0.0174 (10)	
H21	0.7943	0.1451	0.4244	0.021*	
C22	0.5991 (6)	0.2123 (2)	0.43380 (15)	0.0163 (10)	
H22	0.5162	0.1757	0.4315	0.020*	
C23	0.5604 (5)	0.2865 (2)	0.44160 (15)	0.0132 (9)	
C24	0.3991 (6)	0.3206 (2)	0.44915 (15)	0.0146 (9)	
Br2	0.41662 (7)	0.36508 (3)	0.88995 (2)	0.03217 (14)	
O4	0.3369 (4)	0.51090 (15)	0.72672 (11)	0.0169 (7)	
O5	0.2069 (3)	0.69908 (16)	0.79230 (11)	0.0160 (7)	
O6	0.1534 (4)	0.80937 (16)	0.82613 (12)	0.0197 (7)	
N2	0.2868 (5)	0.61160 (19)	0.56403 (13)	0.0177 (9)	
C25	0.3579 (5)	0.5173 (2)	0.77747 (15)	0.0138 (9)	
C26	0.3746 (5)	0.4512 (2)	0.80330 (16)	0.0181 (10)	
H26	0.3753	0.4054	0.7863	0.022*	
C27	0.3900 (6)	0.4545 (2)	0.85438 (17)	0.0216 (11)	
C28	0.3868 (6)	0.5198 (2)	0.88052 (17)	0.0210 (11)	
H28	0.3940	0.5202	0.9158	0.025*	
C29	0.3727 (5)	0.5850 (3)	0.85389 (16)	0.0188 (10)	
H29	0.3722	0.6305	0.8713	0.023*	
C30	0.3594 (5)	0.5848 (2)	0.80207 (16)	0.0147 (9)	
C31	0.3510 (5)	0.6566 (2)	0.77414 (16)	0.0149 (9)	
C32	0.3316 (5)	0.6439 (2)	0.71915 (16)	0.0134 (9)	
C33	0.3218 (5)	0.7029 (2)	0.68601 (17)	0.0167 (10)	
H33	0.3261	0.7515	0.6988	0.020*	
C34	0.3063 (5)	0.6929 (2)	0.63567 (17)	0.0157 (10)	
H34	0.2980	0.7347	0.6146	0.019*	
C35	0.3021 (5)	0.6217 (2)	0.61428 (16)	0.0140 (9)	
C36	0.3144 (5)	0.5622 (2)	0.64719 (16)	0.0155 (10)	
H36	0.3142	0.5134	0.6346	0.019*	
C37	0.3271 (5)	0.5742 (2)	0.69832 (17)	0.0153 (10)	
C38	0.2429 (6)	0.6721 (3)	0.53094 (16)	0.0206 (11)	
H38A	0.1740	0.7075	0.5495	0.025*	
H38B	0.1762	0.6524	0.5032	0.025*	
C39	0.3903 (7)	0.7128 (3)	0.50945 (19)	0.0316 (13)	
H39A	0.4631	0.6776	0.4928	0.047*	
H39B	0.4499	0.7373	0.5364	0.047*	
H39C	0.3525	0.7495	0.4853	0.047*	
C40	0.3094 (5)	0.5399 (2)	0.54013 (16)	0.0157 (10)	
H40A	0.3904	0.5113	0.5596	0.019*	
H40B	0.3559	0.5477	0.5065	0.019*	
C41	0.1539 (6)	0.4950 (3)	0.53531 (18)	0.0235 (11)	
H41A	0.0719	0.5232	0.5167	0.035*	
H41B	0.1113	0.4835	0.5685	0.035*	
H41C	0.1780	0.4491	0.5176	0.035*	
C42	0.4921 (5)	0.7074 (2)	0.78662 (15)	0.0146 (9)	
C43	0.6590 (5)	0.6963 (2)	0.78122 (17)	0.0173 (10)	
H43	0.7009	0.6508	0.7692	0.021*	
C44	0.7637 (6)	0.7538 (3)	0.79392 (16)	0.0202 (11)	
H44	0.8789	0.7471	0.7912	0.024*	
C45	0.7029 (6)	0.8208 (3)	0.81042 (16)	0.0188 (10)	
H45	0.7765	0.8601	0.8171	0.023*	
C46	0.5359 (6)	0.8311 (2)	0.81717 (15)	0.0171 (10)	
H46	0.4936	0.8761	0.8296	0.021*	
C47	0.4327 (5)	0.7732 (2)	0.80510 (15)	0.0140 (10)	
C48	0.2526 (6)	0.7669 (2)	0.80989 (15)	0.0143 (9)	

### (5) (1R)-6'-Bromo-3'-diethylamino-3H-spiro[2-benzofuran-1,9'-xanthen]-3-one Atomic displacement parameters (Å^2^)

**Table:**

	*U*^11^	*U*^22^	*U*^33^	*U*^12^	*U*^13^	*U*^23^
Br1	0.0362 (3)	0.0288 (3)	0.0205 (2)	−0.0066 (2)	−0.0075 (2)	0.0118 (2)
O1	0.0273 (19)	0.0155 (17)	0.0138 (16)	−0.0017 (14)	−0.0018 (14)	0.0019 (13)
O2	0.0097 (16)	0.0177 (15)	0.0185 (16)	0.0001 (12)	0.0024 (13)	0.0030 (12)
O3	0.0149 (18)	0.028 (2)	0.0191 (18)	−0.0049 (15)	−0.0008 (14)	0.0029 (14)
N1	0.018 (2)	0.021 (2)	0.0152 (19)	0.0014 (17)	−0.0011 (16)	0.0002 (16)
C1	0.013 (2)	0.020 (2)	0.015 (2)	0.0021 (19)	−0.0005 (18)	−0.0004 (18)
C2	0.019 (3)	0.014 (2)	0.021 (2)	0.000 (2)	−0.002 (2)	0.0045 (18)
C3	0.016 (3)	0.024 (3)	0.017 (2)	−0.002 (2)	0.0003 (19)	0.0095 (19)
C4	0.019 (3)	0.027 (3)	0.014 (2)	−0.004 (2)	−0.0009 (19)	0.0027 (19)
C5	0.017 (3)	0.016 (2)	0.022 (2)	−0.0027 (19)	0.0017 (19)	0.0015 (19)
C6	0.010 (2)	0.015 (2)	0.019 (2)	0.0011 (19)	0.0011 (19)	0.0013 (18)
C7	0.010 (2)	0.014 (2)	0.017 (2)	−0.0022 (19)	0.0011 (18)	0.0006 (18)
C8	0.011 (2)	0.016 (2)	0.012 (2)	0.0033 (18)	0.0044 (18)	0.0017 (18)
C9	0.015 (2)	0.011 (2)	0.021 (2)	0.0030 (19)	0.0031 (19)	0.0022 (18)
C10	0.015 (2)	0.014 (2)	0.012 (2)	0.0042 (19)	0.0017 (18)	0.0010 (17)
C11	0.010 (2)	0.018 (2)	0.018 (2)	0.0016 (19)	0.0016 (19)	0.0004 (19)
C12	0.013 (2)	0.013 (2)	0.021 (2)	0.0006 (18)	0.0000 (19)	0.0001 (19)
C13	0.013 (2)	0.016 (2)	0.015 (2)	0.0046 (18)	0.0008 (18)	0.0054 (18)
C14	0.023 (3)	0.024 (3)	0.017 (2)	−0.001 (2)	−0.006 (2)	0.000 (2)
C15	0.025 (3)	0.025 (3)	0.016 (2)	−0.003 (2)	0.001 (2)	0.000 (2)
C16	0.022 (2)	0.021 (2)	0.023 (2)	0.000 (2)	0.0003 (19)	−0.001 (2)
C17	0.035 (3)	0.026 (3)	0.030 (3)	0.008 (2)	−0.001 (2)	−0.002 (2)
C18	0.016 (2)	0.015 (2)	0.009 (2)	0.0005 (18)	−0.0009 (17)	0.0006 (17)
C19	0.013 (2)	0.018 (2)	0.014 (2)	−0.0031 (19)	−0.0002 (18)	−0.0012 (18)
C20	0.016 (3)	0.023 (3)	0.015 (2)	0.002 (2)	0.0020 (19)	−0.0012 (19)
C21	0.025 (3)	0.010 (2)	0.018 (2)	0.006 (2)	0.002 (2)	−0.0019 (18)
C22	0.017 (2)	0.016 (2)	0.016 (2)	−0.007 (2)	−0.0005 (19)	−0.0013 (18)
C23	0.010 (2)	0.018 (2)	0.011 (2)	−0.0027 (18)	−0.0023 (17)	0.0008 (17)
C24	0.019 (2)	0.017 (2)	0.009 (2)	−0.002 (2)	−0.0042 (19)	0.0048 (17)
Br2	0.0550 (4)	0.0189 (2)	0.0226 (3)	−0.0016 (3)	−0.0038 (2)	0.0063 (2)
O4	0.0253 (18)	0.0118 (16)	0.0137 (16)	0.0004 (13)	−0.0002 (13)	−0.0007 (12)
O5	0.0130 (16)	0.0137 (16)	0.0214 (18)	0.0008 (13)	0.0012 (13)	−0.0047 (13)
O6	0.0212 (18)	0.0146 (17)	0.0234 (18)	0.0058 (14)	−0.0011 (14)	−0.0038 (13)
N2	0.025 (2)	0.0130 (19)	0.0146 (19)	0.0028 (16)	0.0010 (17)	0.0005 (15)
C25	0.011 (2)	0.019 (2)	0.011 (2)	−0.0026 (18)	0.0023 (18)	−0.0015 (18)
C26	0.023 (3)	0.013 (2)	0.018 (2)	−0.002 (2)	−0.001 (2)	0.0002 (18)
C27	0.024 (3)	0.016 (2)	0.025 (3)	−0.001 (2)	−0.005 (2)	0.002 (2)
C28	0.030 (3)	0.017 (2)	0.016 (2)	−0.001 (2)	−0.003 (2)	0.0015 (18)
C29	0.023 (3)	0.017 (2)	0.017 (2)	−0.003 (2)	−0.002 (2)	−0.0030 (19)
C30	0.012 (2)	0.016 (2)	0.017 (2)	−0.0029 (19)	−0.0013 (18)	−0.0002 (18)
C31	0.011 (2)	0.015 (2)	0.019 (2)	0.0036 (17)	0.0035 (18)	−0.0014 (18)
C32	0.013 (2)	0.012 (2)	0.016 (2)	−0.0023 (18)	−0.0001 (18)	0.0016 (19)
C33	0.014 (2)	0.014 (2)	0.022 (3)	0.0023 (19)	0.002 (2)	0.0011 (19)
C34	0.014 (2)	0.011 (2)	0.022 (2)	0.0005 (19)	−0.0011 (19)	0.0038 (19)
C35	0.011 (2)	0.016 (2)	0.015 (2)	0.0015 (17)	0.0013 (17)	−0.0025 (18)
C36	0.015 (2)	0.013 (2)	0.019 (2)	0.0032 (19)	0.0007 (19)	−0.0016 (19)
C37	0.011 (2)	0.015 (2)	0.020 (2)	−0.0014 (19)	0.0010 (18)	0.0013 (19)
C38	0.027 (3)	0.021 (3)	0.014 (2)	0.004 (2)	−0.003 (2)	0.0025 (19)
C39	0.040 (3)	0.023 (3)	0.032 (3)	0.001 (3)	0.011 (3)	0.006 (2)
C40	0.014 (2)	0.020 (2)	0.013 (2)	0.004 (2)	−0.0001 (18)	−0.0015 (18)
C41	0.019 (3)	0.024 (3)	0.027 (3)	0.001 (2)	0.001 (2)	−0.004 (2)
C42	0.018 (2)	0.012 (2)	0.014 (2)	−0.0009 (18)	−0.0014 (19)	−0.0008 (18)
C43	0.017 (2)	0.020 (2)	0.015 (2)	0.0016 (19)	0.000 (2)	−0.001 (2)
C44	0.017 (3)	0.025 (3)	0.019 (3)	−0.002 (2)	−0.0004 (19)	0.003 (2)
C45	0.020 (3)	0.021 (3)	0.016 (2)	−0.007 (2)	−0.0051 (19)	0.0021 (19)
C46	0.026 (3)	0.013 (2)	0.013 (2)	−0.001 (2)	−0.002 (2)	0.0020 (18)
C47	0.018 (3)	0.015 (2)	0.009 (2)	−0.0012 (19)	−0.0036 (18)	0.0028 (17)
C48	0.019 (2)	0.016 (2)	0.008 (2)	0.004 (2)	−0.0014 (18)	0.0019 (18)

### (5) (1R)-6'-Bromo-3'-diethylamino-3H-spiro[2-benzofuran-1,9'-xanthen]-3-one Geometric parameters (Å, º)

**Table:**

Br1—C3	1.893 (4)	Br2—C27	1.899 (4)
O1—C1	1.373 (5)	O4—C25	1.379 (5)
O1—C13	1.389 (5)	O4—C37	1.383 (5)
O2—C24	1.379 (5)	O5—C48	1.373 (5)
O2—C7	1.510 (5)	O5—C31	1.488 (5)
O3—C24	1.201 (5)	O6—C48	1.200 (5)
N1—C11	1.379 (5)	N2—C35	1.368 (5)
N1—C14	1.455 (5)	N2—C38	1.458 (5)
N1—C16	1.466 (6)	N2—C40	1.465 (5)
C1—C6	1.376 (6)	C25—C26	1.394 (6)
C1—C2	1.394 (6)	C25—C30	1.394 (6)
C2—C3	1.374 (6)	C26—C27	1.379 (6)
C2—H2	0.9500	C26—H26	0.9500
C3—C4	1.390 (6)	C27—C28	1.380 (6)
C4—C5	1.383 (6)	C28—C29	1.388 (6)
C4—H4	0.9500	C28—H28	0.9500
C5—C6	1.406 (6)	C29—C30	1.396 (6)
C5—H5	0.9500	C29—H29	0.9500
C6—C7	1.516 (6)	C30—C31	1.508 (6)
C7—C8	1.499 (6)	C31—C32	1.503 (6)
C7—C18	1.499 (6)	C31—C42	1.513 (6)
C8—C13	1.376 (6)	C32—C37	1.387 (6)
C8—C9	1.403 (6)	C32—C33	1.395 (6)
C9—C10	1.371 (6)	C33—C34	1.370 (6)
C9—H9	0.9500	C33—H33	0.9500
C10—C11	1.423 (6)	C34—C35	1.418 (6)
C10—H10	0.9500	C34—H34	0.9500
C11—C12	1.403 (6)	C35—C36	1.401 (6)
C12—C13	1.391 (6)	C36—C37	1.394 (6)
C12—H12	0.9500	C36—H36	0.9500
C14—C15	1.522 (6)	C38—C39	1.525 (7)
C14—H14A	0.9900	C38—H38A	0.9900
C14—H14B	0.9900	C38—H38B	0.9900
C15—H15A	0.9800	C39—H39A	0.9800
C15—H15B	0.9800	C39—H39B	0.9800
C15—H15C	0.9800	C39—H39C	0.9800
C16—C17	1.524 (6)	C40—C41	1.513 (6)
C16—H16A	0.9900	C40—H40A	0.9900
C16—H16B	0.9900	C40—H40B	0.9900
C17—H17A	0.9800	C41—H41A	0.9800
C17—H17B	0.9800	C41—H41B	0.9800
C17—H17C	0.9800	C41—H41C	0.9800
C18—C23	1.377 (6)	C42—C47	1.382 (6)
C18—C19	1.398 (6)	C42—C43	1.384 (6)
C19—C20	1.376 (6)	C43—C44	1.393 (6)
C19—H19	0.9500	C43—H43	0.9500
C20—C21	1.404 (6)	C44—C45	1.388 (6)
C20—H20	0.9500	C44—H44	0.9500
C21—C22	1.378 (6)	C45—C46	1.386 (6)
C21—H21	0.9500	C45—H45	0.9500
C22—C23	1.402 (6)	C46—C47	1.386 (6)
C22—H22	0.9500	C46—H46	0.9500
C23—C24	1.467 (6)	C47—C48	1.478 (6)
			
C1—O1—C13	118.4 (3)	C25—O4—C37	118.8 (3)
C24—O2—C7	110.6 (3)	C48—O5—C31	111.4 (3)
C11—N1—C14	120.6 (4)	C35—N2—C38	121.5 (4)
C11—N1—C16	120.7 (4)	C35—N2—C40	122.7 (3)
C14—N1—C16	118.1 (4)	C38—N2—C40	115.8 (4)
O1—C1—C6	123.5 (4)	O4—C25—C26	115.6 (4)
O1—C1—C2	114.9 (4)	O4—C25—C30	122.9 (4)
C6—C1—C2	121.6 (4)	C26—C25—C30	121.5 (4)
C3—C2—C1	118.7 (4)	C27—C26—C25	117.8 (4)
C3—C2—H2	120.7	C27—C26—H26	121.1
C1—C2—H2	120.7	C25—C26—H26	121.1
C2—C3—C4	121.7 (4)	C26—C27—C28	122.8 (4)
C2—C3—Br1	118.9 (3)	C26—C27—Br2	118.3 (3)
C4—C3—Br1	119.4 (3)	C28—C27—Br2	118.9 (3)
C5—C4—C3	118.6 (4)	C27—C28—C29	118.3 (4)
C5—C4—H4	120.7	C27—C28—H28	120.9
C3—C4—H4	120.7	C29—C28—H28	120.9
C4—C5—C6	121.1 (4)	C28—C29—C30	121.2 (4)
C4—C5—H5	119.4	C28—C29—H29	119.4
C6—C5—H5	119.4	C30—C29—H29	119.4
C1—C6—C5	118.2 (4)	C25—C30—C29	118.4 (4)
C1—C6—C7	121.3 (4)	C25—C30—C31	121.8 (4)
C5—C6—C7	120.2 (4)	C29—C30—C31	119.8 (4)
C8—C7—C18	113.9 (4)	O5—C31—C32	108.6 (3)
C8—C7—O2	109.8 (3)	O5—C31—C30	108.8 (3)
C18—C7—O2	101.8 (3)	C32—C31—C30	111.1 (3)
C8—C7—C6	110.6 (3)	O5—C31—C42	102.2 (3)
C18—C7—C6	115.5 (3)	C32—C31—C42	113.0 (3)
O2—C7—C6	104.3 (3)	C30—C31—C42	112.6 (3)
C13—C8—C9	115.8 (4)	C37—C32—C33	116.3 (4)
C13—C8—C7	122.7 (4)	C37—C32—C31	122.6 (4)
C9—C8—C7	121.4 (4)	C33—C32—C31	121.0 (4)
C10—C9—C8	122.7 (4)	C34—C33—C32	122.2 (4)
C10—C9—H9	118.7	C34—C33—H33	118.9
C8—C9—H9	118.7	C32—C33—H33	118.9
C9—C10—C11	120.7 (4)	C33—C34—C35	121.5 (4)
C9—C10—H10	119.6	C33—C34—H34	119.2
C11—C10—H10	119.6	C35—C34—H34	119.2
N1—C11—C12	121.1 (4)	N2—C35—C36	121.7 (4)
N1—C11—C10	121.7 (4)	N2—C35—C34	121.6 (4)
C12—C11—C10	117.1 (4)	C36—C35—C34	116.6 (4)
C13—C12—C11	119.8 (4)	C37—C36—C35	120.4 (4)
C13—C12—H12	120.1	C37—C36—H36	119.8
C11—C12—H12	120.1	C35—C36—H36	119.8
C8—C13—O1	122.2 (4)	O4—C37—C32	122.5 (4)
C8—C13—C12	123.8 (4)	O4—C37—C36	114.7 (4)
O1—C13—C12	114.0 (4)	C32—C37—C36	122.8 (4)
N1—C14—C15	112.9 (4)	N2—C38—C39	113.8 (4)
N1—C14—H14A	109.0	N2—C38—H38A	108.8
C15—C14—H14A	109.0	C39—C38—H38A	108.8
N1—C14—H14B	109.0	N2—C38—H38B	108.8
C15—C14—H14B	109.0	C39—C38—H38B	108.8
H14A—C14—H14B	107.8	H38A—C38—H38B	107.7
C14—C15—H15A	109.5	C38—C39—H39A	109.5
C14—C15—H15B	109.5	C38—C39—H39B	109.5
H15A—C15—H15B	109.5	H39A—C39—H39B	109.5
C14—C15—H15C	109.5	C38—C39—H39C	109.5
H15A—C15—H15C	109.5	H39A—C39—H39C	109.5
H15B—C15—H15C	109.5	H39B—C39—H39C	109.5
N1—C16—C17	114.1 (4)	N2—C40—C41	114.3 (4)
N1—C16—H16A	108.7	N2—C40—H40A	108.7
C17—C16—H16A	108.7	C41—C40—H40A	108.7
N1—C16—H16B	108.7	N2—C40—H40B	108.7
C17—C16—H16B	108.7	C41—C40—H40B	108.7
H16A—C16—H16B	107.6	H40A—C40—H40B	107.6
C16—C17—H17A	109.5	C40—C41—H41A	109.5
C16—C17—H17B	109.5	C40—C41—H41B	109.5
H17A—C17—H17B	109.5	H41A—C41—H41B	109.5
C16—C17—H17C	109.5	C40—C41—H41C	109.5
H17A—C17—H17C	109.5	H41A—C41—H41C	109.5
H17B—C17—H17C	109.5	H41B—C41—H41C	109.5
C23—C18—C19	120.7 (4)	C47—C42—C43	120.6 (4)
C23—C18—C7	110.5 (4)	C47—C42—C31	110.0 (4)
C19—C18—C7	128.8 (4)	C43—C42—C31	129.4 (4)
C20—C19—C18	117.5 (4)	C42—C43—C44	117.8 (4)
C20—C19—H19	121.2	C42—C43—H43	121.1
C18—C19—H19	121.2	C44—C43—H43	121.1
C19—C20—C21	121.7 (4)	C45—C44—C43	121.3 (4)
C19—C20—H20	119.2	C45—C44—H44	119.4
C21—C20—H20	119.2	C43—C44—H44	119.4
C22—C21—C20	121.0 (4)	C46—C45—C44	120.7 (4)
C22—C21—H21	119.5	C46—C45—H45	119.7
C20—C21—H21	119.5	C44—C45—H45	119.7
C21—C22—C23	116.9 (4)	C47—C46—C45	117.6 (4)
C21—C22—H22	121.5	C47—C46—H46	121.2
C23—C22—H22	121.5	C45—C46—H46	121.2
C18—C23—C22	122.1 (4)	C42—C47—C46	121.9 (4)
C18—C23—C24	108.9 (4)	C42—C47—C48	108.2 (4)
C22—C23—C24	129.0 (4)	C46—C47—C48	129.9 (4)
O3—C24—O2	121.1 (4)	O6—C48—O5	121.3 (4)
O3—C24—C23	131.2 (4)	O6—C48—C47	130.7 (4)
O2—C24—C23	107.7 (4)	O5—C48—C47	108.0 (4)
			
C13—O1—C1—C6	3.3 (6)	C37—O4—C25—C26	−177.1 (4)
C13—O1—C1—C2	−177.9 (4)	C37—O4—C25—C30	4.2 (6)
O1—C1—C2—C3	−179.0 (4)	O4—C25—C26—C27	−177.7 (4)
C6—C1—C2—C3	−0.2 (7)	C30—C25—C26—C27	1.1 (6)
C1—C2—C3—C4	−2.1 (7)	C25—C26—C27—C28	1.1 (7)
C1—C2—C3—Br1	177.4 (3)	C25—C26—C27—Br2	−178.9 (3)
C2—C3—C4—C5	1.8 (7)	C26—C27—C28—C29	−2.1 (7)
Br1—C3—C4—C5	−177.7 (3)	Br2—C27—C28—C29	177.9 (3)
C3—C4—C5—C6	0.8 (7)	C27—C28—C29—C30	1.0 (7)
O1—C1—C6—C5	−178.7 (4)	O4—C25—C30—C29	176.6 (4)
C2—C1—C6—C5	2.7 (7)	C26—C25—C30—C29	−2.1 (6)
O1—C1—C6—C7	7.1 (7)	O4—C25—C30—C31	−4.8 (6)
C2—C1—C6—C7	−171.5 (4)	C26—C25—C30—C31	176.5 (4)
C4—C5—C6—C1	−3.0 (7)	C28—C29—C30—C25	1.0 (7)
C4—C5—C6—C7	171.3 (4)	C28—C29—C30—C31	−177.6 (4)
C24—O2—C7—C8	−128.9 (4)	C48—O5—C31—C32	−116.1 (4)
C24—O2—C7—C18	−7.9 (4)	C48—O5—C31—C30	122.8 (3)
C24—O2—C7—C6	112.6 (3)	C48—O5—C31—C42	3.5 (4)
C1—C6—C7—C8	−12.5 (6)	C25—C30—C31—O5	122.8 (4)
C5—C6—C7—C8	173.4 (4)	C29—C30—C31—O5	−58.7 (5)
C1—C6—C7—C18	−143.7 (4)	C25—C30—C31—C32	3.3 (5)
C5—C6—C7—C18	42.2 (5)	C29—C30—C31—C32	−178.2 (4)
C1—C6—C7—O2	105.5 (4)	C25—C30—C31—C42	−124.7 (4)
C5—C6—C7—O2	−68.6 (5)	C29—C30—C31—C42	53.8 (5)
C18—C7—C8—C13	141.0 (4)	O5—C31—C32—C37	−121.2 (4)
O2—C7—C8—C13	−105.6 (4)	C30—C31—C32—C37	−1.6 (5)
C6—C7—C8—C13	9.0 (6)	C42—C31—C32—C37	126.2 (4)
C18—C7—C8—C9	−39.6 (5)	O5—C31—C32—C33	60.6 (5)
O2—C7—C8—C9	73.7 (5)	C30—C31—C32—C33	−179.8 (4)
C6—C7—C8—C9	−171.7 (4)	C42—C31—C32—C33	−52.0 (5)
C13—C8—C9—C10	−0.3 (6)	C37—C32—C33—C34	0.9 (6)
C7—C8—C9—C10	−179.7 (4)	C31—C32—C33—C34	179.1 (4)
C8—C9—C10—C11	−1.4 (7)	C32—C33—C34—C35	−1.1 (7)
C14—N1—C11—C12	174.3 (4)	C38—N2—C35—C36	168.1 (4)
C16—N1—C11—C12	2.9 (6)	C40—N2—C35—C36	−10.5 (6)
C14—N1—C11—C10	−9.5 (6)	C38—N2—C35—C34	−11.8 (6)
C16—N1—C11—C10	179.0 (4)	C40—N2—C35—C34	169.5 (4)
C9—C10—C11—N1	−173.3 (4)	C33—C34—C35—N2	−179.9 (4)
C9—C10—C11—C12	3.0 (6)	C33—C34—C35—C36	0.1 (6)
N1—C11—C12—C13	173.2 (4)	N2—C35—C36—C37	−178.9 (4)
C10—C11—C12—C13	−3.1 (6)	C34—C35—C36—C37	1.1 (6)
C9—C8—C13—O1	−179.1 (4)	C25—O4—C37—C32	−2.4 (6)
C7—C8—C13—O1	0.3 (6)	C25—O4—C37—C36	176.8 (4)
C9—C8—C13—C12	0.2 (6)	C33—C32—C37—O4	179.5 (4)
C7—C8—C13—C12	179.6 (4)	C31—C32—C37—O4	1.3 (6)
C1—O1—C13—C8	−7.1 (6)	C33—C32—C37—C36	0.4 (6)
C1—O1—C13—C12	173.5 (4)	C31—C32—C37—C36	−177.9 (4)
C11—C12—C13—C8	1.6 (7)	C35—C36—C37—O4	179.4 (4)
C11—C12—C13—O1	−179.1 (4)	C35—C36—C37—C32	−1.4 (7)
C11—N1—C14—C15	−77.4 (5)	C35—N2—C38—C39	91.9 (5)
C16—N1—C14—C15	94.2 (5)	C40—N2—C38—C39	−89.4 (5)
C11—N1—C16—C17	76.2 (5)	C35—N2—C40—C41	90.8 (5)
C14—N1—C16—C17	−95.4 (5)	C38—N2—C40—C41	−87.9 (5)
C8—C7—C18—C23	122.5 (4)	O5—C31—C42—C47	−5.1 (4)
O2—C7—C18—C23	4.4 (4)	C32—C31—C42—C47	111.4 (4)
C6—C7—C18—C23	−107.9 (4)	C30—C31—C42—C47	−121.7 (4)
C8—C7—C18—C19	−54.7 (6)	O5—C31—C42—C43	175.9 (4)
O2—C7—C18—C19	−172.8 (4)	C32—C31—C42—C43	−67.6 (6)
C6—C7—C18—C19	74.9 (6)	C30—C31—C42—C43	59.3 (6)
C23—C18—C19—C20	0.8 (6)	C47—C42—C43—C44	−1.4 (7)
C7—C18—C19—C20	177.7 (4)	C31—C42—C43—C44	177.5 (4)
C18—C19—C20—C21	−0.7 (6)	C42—C43—C44—C45	−1.4 (7)
C19—C20—C21—C22	0.4 (7)	C43—C44—C45—C46	3.4 (7)
C20—C21—C22—C23	−0.2 (6)	C44—C45—C46—C47	−2.4 (6)
C19—C18—C23—C22	−0.7 (6)	C43—C42—C47—C46	2.4 (7)
C7—C18—C23—C22	−178.1 (4)	C31—C42—C47—C46	−176.7 (4)
C19—C18—C23—C24	177.7 (4)	C43—C42—C47—C48	−176.1 (4)
C7—C18—C23—C24	0.3 (5)	C31—C42—C47—C48	4.8 (5)
C21—C22—C23—C18	0.4 (6)	C45—C46—C47—C42	−0.4 (6)
C21—C22—C23—C24	−177.7 (4)	C45—C46—C47—C48	177.6 (4)
C7—O2—C24—O3	−171.6 (4)	C31—O5—C48—O6	179.9 (4)
C7—O2—C24—C23	8.4 (4)	C31—O5—C48—C47	−0.9 (4)
C18—C23—C24—O3	174.5 (4)	C42—C47—C48—O6	176.6 (4)
C22—C23—C24—O3	−7.2 (8)	C46—C47—C48—O6	−1.7 (8)
C18—C23—C24—O2	−5.4 (5)	C42—C47—C48—O5	−2.5 (5)
C22—C23—C24—O2	172.8 (4)	C46—C47—C48—O5	179.2 (4)

### (5) (1R)-6'-Bromo-3'-diethylamino-3H-spiro[2-benzofuran-1,9'-xanthen]-3-one Hydrogen-bond geometry (Å, º)

Cg1 and Cg2 are the centroids of the C8–C13 and O1,C1,C6,C7,C8,C13 rings, respectively.

**Table:**

*D*—H···*A*	*D*—H	H···*A*	*D*···*A*	*D*—H···*A*
C16—H16*B*···*Cg*^i^	0.99	2.81	3.583 (4)	136
C40—H40*A*···*Cg*1	0.99	2.79	3.534 (4)	132
C40—H40*B*···*Cg*2	0.99	2.83	3.580 (4)	133

Symmetry code: (i) *x*+1, *y*, *z*.

## References

[bb1] Berscheid, R., Nieger, M. & Vögtle, F. (1992). *Chem. Ber.* **125**, 2539–2552.

[bb2] Brandenburg, K. & Putz, H. (2012). *DIAMOND*. Crystal Impact GbR, Bonn, Germany.

[bb3] Bruker (2016). *APEX3*, *SADABS* and *SAINT*. Bruker AXS Inc., Madison, Wisconsin, USA.

[bb4] Bučar, D.-K., Filip, S., Arhangelskis, M., Lloyd, G. O. & Jones, W. (2013). *CrystEngComm*, **15**, 6289–6291.

[bb5] Chen, X. Q., Pradhan, T., Wang, F., Kim, J. S. & Yoon, J. (2012). *Chem. Rev.* **112**, 1910–1956. 10.1021/cr200201z 22040233

[bb6] Hou, F., Cheng, J., Xi, P., Chen, F., Huang, L., Xie, G., Shi, Y., Liu, H., Bai, D. & Zeng, Z. (2012). *Dalton Trans.* **41**, 5799–5804. 10.1039/c2dt12462a 22437757

[bb7] Jo, H. H., Lin, C. Y. & Anslyn, E. V. (2014). *Acc. Chem. Res.* **47**, 2212–2221. 10.1021/ar500147x 24892802

[bb8] Kvick, Å., Vaughan, G. B. M., Wang, X., Sun, Y. & Long, Y. (2000). *Acta Cryst.* C**56**, 1232–1233. 10.1107/s0108270100008404 11025307

[bb9] LaPlante, S. R. F., Fader, L. D., Fandrick, K. R., Fandrick, D. R., Hucke, O., Kemper, R., Miller, S. P. F. & Edwards, P. J. (2011). *J. Med. Chem.* **54**, 7005–7022. 10.1021/jm200584g 21848318

[bb10] Li, X. M., Ding, C. F., Tian, B. Q., Liu, Q., Zhang, S. S., Xu, H. & Ouyang, P. K. (2006). *Chem. Pap.* **60**, 220–223.

[bb11] Liu, X.-L., Wang, J.-L., Liu, J.-W. & Miao, F.-M. (1995). *Acta Cryst.* C**51**, 324–326.

[bb12] Mchedlov-Petrossyan, N. O., Cheipesh, T. A., Shekhovtsov, S. V., Redko, A. N., Rybachenko, V. I., Omelchenko, I. V. & Shishkin, O. V. (2015). *Spectrochim. Acta Part A*, **150**, 151–161. 10.1016/j.saa.2015.05.037 26037500

[bb13] Miao, F.-M., Zhang, L.-J., Wen, X., Zhou, W.-H., Niu, Z.-C., Han, J.-G. & Liu, X.-L. (1996). *Acta Cryst.* C**52**, 700–702.

[bb14] Okada, K. (1996). *J. Mol. Struct.* **380**, 235–247.

[bb15] Pak, Y. L., Swamy, K. M. & Yoon, J. (2015). *Sensors (Basel)*, **15**, 24374–24396. 10.3390/s150924374 PMC4610470 26402684

[bb16] Parsons, S., Flack, H. D. & Wagner, T. (2013). *Acta Cryst.* B**69**, 249–259. 10.1107/S2052519213010014 PMC3661305 23719469

[bb17] Reist, M., Carrupt, P. A., Francotte, E. & Testa, B. (1998). *Chem. Res. Toxicol.* **11**, 1521–1528. 10.1021/tx9801817 9860497

[bb18] Sheldrick, G. M. (2008). *Acta Cryst.* A**64**, 112–122. 10.1107/S0108767307043930 18156677

[bb19] Sheldrick, G. M. (2015*a*). *Acta Cryst.* A**71**, 3–8.

[bb20] Sheldrick, G. M. (2015*b*). *Acta Cryst.* C**71**, 3–8.

[bb21] Shimizu, K. D. & Stephenson, C. J. (2010). *Curr. Opin. Chem. Biol.* **14**, 743–750. 10.1016/j.cbpa.2010.07.007 20685156

[bb22] Swamy, K. M. K., Kim, H. N., Soh, J. H., Kim, Y., Kim, S.-J. & Yoon, J. (2009). *Chem. Commun.* pp. 1234–1236. 10.1039/b819538b 19240884

[bb23] Wang, M., Marriott, P. J., Chan, W. H., Lee, A. W. M. & Huie, C. W. (2006). *J. Chromatogr. A*, **1112**, 361–368. 10.1016/j.chroma.2005.12.043 16387317

[bb24] Wang, W., Rusin, O., Xu, X., Kim, K. K., Escobedo, J. O., Fakayode, S. O., Fletcher, K. A., Lowry, M., Schowalter, C. M., Lawrence, C. M., Fronczek, F. R., Warner, I. M. & Strongin, R. M. (2005). *J. Am. Chem. Soc.* **127**, 15949–15958. 10.1021/ja054962n PMC3386615 16277539

[bb25] Wang, L.-F., Wang, X., Peng, Z., He, F. & Wang, Q. (1990). *Acta Cryst.* C**46**, 1676–1678.

[bb26] You, L., Zha, D. & Anslyn, E. V. (2015). *Chem. Rev.* **115**, 7840–7892. 10.1021/cr5005524 25719867

[bb27] Yu, S. & Pu, L. (2015). *Tetrahedron*, **71**, 745–772.

[bb28] Zhang, I., Wang, Y., Wan, C., Xing, Z., Li, W., Li, M. & Zhang, S. X.-A. (2015). *RSC Adv.* **5**, 66416–66419.

[bb29] Zhang, X., Yin, J. & Yoon, J. (2014). *Chem. Rev.* **114**, 4918–4959. 10.1021/cr400568b 24499128

